# Genome-Wide Association Study for Identifying Loci that Affect Fillet Yield, Carcass, and Body Weight Traits in Rainbow Trout (*Oncorhynchus mykiss*)

**DOI:** 10.3389/fgene.2016.00203

**Published:** 2016-11-22

**Authors:** Dianelys Gonzalez-Pena, Guangtu Gao, Matthew Baranski, Thomas Moen, Beth M. Cleveland, P. Brett Kenney, Roger L. Vallejo, Yniv Palti, Timothy D. Leeds

**Affiliations:** ^1^United States Department of Agriculture, National Center for Cool and Cold Water Aquaculture, Agricultural Research ServiceKearneysville, WV, USA; ^2^NofimaÅs, Norway; ^3^AquagenÅs, Norway; ^4^Division of Animal and Nutritional Sciences, West Virginia UniversityMorgantown, WV, USA

**Keywords:** fillet yield, gene network, genome-wide association study, genomic selection, linkage map, rainbow trout, single-step GBLUP

## Abstract

Fillet yield (FY, %) is an economically-important trait in rainbow trout aquaculture that affects production efficiency. Despite that, FY has received little attention in breeding programs because it is difficult to measure on a large number of fish and cannot be directly measured on breeding candidates. The recent development of a high-density SNP array for rainbow trout has provided the needed tool for studying the underlying genetic architecture of this trait. A genome-wide association study (GWAS) was conducted for FY, body weight at 10 (BW10) and 13 (BW13) months post-hatching, head-off carcass weight (CAR), and fillet weight (FW) in a pedigreed rainbow trout population selectively bred for improved growth performance. The GWAS analysis was performed using the weighted single-step GBLUP method (wssGWAS). Phenotypic records of 1447 fish (1.5 kg at harvest) from 299 full-sib families in three successive generations, of which 875 fish from 196 full-sib families were genotyped, were used in the GWAS analysis. A total of 38,107 polymorphic SNPs were analyzed in a univariate model with hatch year and harvest group as fixed effects, harvest weight as a continuous covariate, and animal and common environment as random effects. A new linkage map was developed to create windows of 20 adjacent SNPs for use in the GWAS. The two windows with largest effect for FY and FW were located on chromosome Omy9 and explained only 1.0–1.5% of genetic variance, thus suggesting a polygenic architecture affected by multiple loci with small effects in this population. One window on Omy5 explained 1.4 and 1.0% of the genetic variance for BW10 and BW13, respectively. Three windows located on Omy27, Omy17, and Omy9 (same window detected for FY) explained 1.7, 1.7, and 1.0%, respectively, of genetic variance for CAR. Among the detected 100 SNPs, 55% were located directly in genes (intron and exons). Nucleotide sequences of intragenic SNPs were blasted to the *Mus musculus* genome to create a putative gene network. The network suggests that differences in the ability to maintain a proliferative and renewable population of myogenic precursor cells may affect variation in growth and fillet yield in rainbow trout.

## Introduction

Fillet yield (FY, %), the ratio between the separable muscle of the fish and the harvest weight, is an economically important trait in rainbow trout aquaculture that reflects production efficiency with a consequent large impact on the returns (Kause et al., 2002). On a per-unit basis, the fillet price is three times that of the whole fish (Bugeon et al., 2010) and a recent global survey ranked FY among the six most important traits for genetic improvement in rainbow trout aquaculture (Sae-Lim et al., 2012). Despite that, FY has not received much attention in breeding programs because it is a lethally-measured trait, costly to measure, and difficult to select on, thus limiting the genetic progress in traditional selective breeding programs (Kause et al., 2007).

To overcome these phenotyping constraints and to increase the accuracy of selection, the use of high-density single nucleotide polymorphism (SNP) genotyping platforms and novel statistical models can identify SNPs associated with the trait for use in genomic selection (Dekkers, 2012). Several genome-wide association studies (GWAS) that match genetic variants, with or without pedigree records, with the observed phenotype have identified significant loci associated with economically important traits in livestock (Kadarmideen, 2014; Sharma et al., 2015). The aquaculture research community is following this trend, and interesting examples are available for growth and disease resistance traits in Atlantic Salmon (e.g., Correa et al., 2015; Gutierrez et al., 2015), rainbow trout (e.g., Kocmarek et al., 2015; Liu et al., 2015b; Palti et al., 2015b), and catfish (e.g., Geng et al., 2015).

Single-step genomic best linear unbiased prediction (ssGBLUP) and Bayesian variable selection methods are available for genomic predictions using dense SNP arrays. The ssGBLUP method (Misztal et al., 2009; Christensen and Lund, 2010) jointly incorporates all available genotypic, phenotypic, and pedigree data in the genomic predictions, but the method's assumption of an infinitesimal model is violated for traits affected by major genes. In contrast, Bayesian variable selection methods assume a prior distribution of the variance associated with each locus (Meuwissen et al., 2001), but are limited in that only data from genotyped animals are used in the analyses. Despite the limitations on data, the Bayesian variable selection method has resulted in greater accuracy of predicted genetic merit compared to ssGBLUP for traits known to have QTL segregating with moderate or large effect like bacterial cold water disease resistance in rainbow trout (Vallejo et al., submitted manuscript). The ssGBLUP method has been extended to a weighted ssGBLUP (wssGBLUP) that emulates the Bayesian variable selection method by allowing for unequal variances across loci (Wang et al., 2012).

In rainbow trout, the recent development of the 57K SNP Axiom® Trout Genotyping Array (Palti et al., 2015a; Affymetrix, Santa Clara, CA), a new genetic linkage map (published here) developed using 47,939 markers included in the 57K SNP Axiom® Trout Genotyping Array, and the release of a reference genome (Berthelot et al., 2014) have provided tools for studying FY using GWAS. The objectives of this study were to conduct a GWAS for FY and other carcass and body weight traits in a rainbow trout population developed at the National Center for Cool and Cold Water Aquaculture (NCCCWA) and selectively bred for improved growth performance, and to visualize gene networks and functional categories enriched among the putative genes detected by GWAS for the studied traits.

## Materials and methods

### Ethics statement

The National Center for Cool and Cold Water Aquaculture (NCCCWA) Institutional Animal Care and Use Committee (Leetown, WV) reviewed and approved all experimental procedures used in this study (Protocol #056).

### NCCCWA population

The development, husbandry practices, and selective breeding of the resource population used in this study have been previously described (Leeds et al., 2016). Briefly, the population was initially developed by intercrossing 7 domesticated strains of rainbow trout. The population was closed to outside germplasm in 2004, and has since been selectively bred each generation for improved growth performance using an index of 10-month body weight and thermal growth coefficient for the growth period between 10 and 13 months of age. Full-sib families characterized for harvest traits in the current study were from the third (2010 year class; 98 families), fourth (2012 year class; 99 families), and fifth (2014 year class; 102 families) generations of selection. The population was converted to all-female families beginning in 2010 by using masculinized females as sires, thus the proportion of males was only 11% in 2010, 4% in 2012, and 0.6% in 2014. All data used in the current study were from non-masculinized fish. All families were maintained in separate tanks to retain pedigree information until tagging with a passive integrated transponder (Avid Identifiction Systems Inc., Norco, CA) at 4–5 months post-hatch. An average of 15.6 fish per family was tagged in 2010, 2012, and 2014 (range = 8–17 fish per family). After tagging, fish from all families were commingled in replicate grow-out tanks. All fish were fed a standard commercial diet (Ziegler Bros, Inc., Gardners, PA) throughout the study using automated feeders (Arvotec, Huutokoski, Finland).

### Traits

Individual body weight data at 10 months (BW10; 295 d ± 18.8) and 13 months (BW13; 386 d ± 12.9) post-hatch were recorded as part of the selective breeding program (Leeds et al., 2016) and were available from 2002 to 2014 (Table 1).

**Table 1:** **Number of observation (n), mean, standard deviation (SD), and coefficient of variation (CV, %) of the analyzed traits and weight at harvest**.

**Trait**	**Acronym**	**Trait type**	**N**	**Mean**	**SD**	**CV (%)**
10-month BW, g	BW10	Biometric	17,174	416.5	153.7	36.9
13-month BW, g	BW13	Biometric	15,810	896.3	328.8	36.7
Weight at harvest, g	Co-variable	Biometric	1447	1472.7	438.4	29.8
Eviscerated BW without head, g	CAR	Biometric	1445	1122.9	336.6	30.0
Weight of fillet, g	FW	Biometric	1447	739.4	235.2	31.8
Weight of fillet/Weight at harvest (%)	FY	Yield	1447	49.82	2.7	5.4

Five fish from each 2010, 2012, and 2014 year class full-sib family were identified for characterization of FY. The aim was to sample fish that represented the range of body weights within each family, with the exception that the largest and smallest fish from each family were excluded. Most families had 15 fish available for sampling at 13 months of age (range = 8–17 fish per family). Thus, to identify fish for FY characterization, the dataset was sorted by family and in descending order of body weight and every 2nd or 3rd fish was selected so that the distribution of body weights was uniformly centered around the median of the family. Selected fish were assigned to one of five harvest groups each generation (~100 fish per harvest group) with the aim of having one fish per family per harvest group. One harvest group per week was processed in each of five consecutive weeks, with the exception that year class 2012 fish were harvested over a 6-week period due to scheduling conflicts. Fish were taken off feed 5 days before harvesting. Fish were harvested between 410 and 437 days post-hatch (mean body weight = 985 g; *SD* = 239 g), between 446 and 481 days post-hatch (mean body weight = 1803 g; *SD* = 305 g), and between 407 and 435 days post-hatch (mean body weight = 1617 g; *SD* = 255 g) for the 2010, 2012, and 2014 hatch years, respectively. At harvest, fish were euthanized using a lethal dose of tricaine methanesulfonate (Tricaine-S, Western Chemical, Ferndale, WA), weighed, eviscerated, and placed on ice overnight. The next day, carcasses were beheaded, weighed, and hand-filleted by a single, experienced technician. The same technician filleted all fish from the 2010 and 2012 year class families, and a different technician filleted all fish from the 2014 year class families. Fillet weight was recorded as the sum of both fillets for each fish; fillet weights excluded the skin in 2010 and 2012 year class families but included skin in 2014 year class families. A summary of the records available, mean, standard deviation and coefficient of variation for each trait is presented in Table 1.

### Genetic linkage map

As the current rainbow trout refrence genome (Berthelot et al., 2014) is fragmented into sequence scaffolds and true chromosome sequences are not yet available as a reference for genetic analyses like GWAS, we generated a new dense linkage map which was used as a genetic map reference in this study (Table S1). The 57K SNP Axiom® Trout Genotyping Array (Palti et al., 2015a) was used to genotype (GeneSeek, Inc., Lincoln, NE) 2464 samples collected across 46 full-sib families from a commercial Norwegian population and 10 full-sib families from the NCCCWA odd-year breeding population. Following quality control of raw genotype data as previously described (Palti et al., 2015a), linkage mapping was performed with Lep-MAP software (Rastas et al., 2013). First, SNPs were assigned to linkage groups with the “SeparateChromosomes” command using increasing LOD thresholds until the observed number of linkage groups corresponded with the haploid chromosome number in this species. Additional SNPs were subsequently added to the groups with the “JoinSingles” command at a more relaxed LOD threshold, and finally SNPs were ordered in each linkage group with the “OrderMarkers” command. Numerous iterations were performed to optimize error and recombination parameters. A total of 47,839 SNPs were mapped to 29 linkage groups, with an average of 1650 SNPs per group. The number of SNPs assigned to each group ranged from 754 to 2934. The total distances covered by the male and female maps were 2214 cM and 4248 cM, respectively. In all 13 chromosomes, known to have homologous pairing with at least one other chromosome arm, female/male recombination ratios were >2.0; whereas, in non-duplicated chromosomes, the female/male recombination ratio ranged from 1.0 to 2.0, with the exception of chromosomes Omy15 and Omy21.

### Genotyping for FY GWAS

The 57K SNP Axiom® Trout Genotyping Array (Palti et al., 2015a) was used to genotype 941 fish with FY phenotypes from the 2010 and 2012 year classes (197 full-sib families) and 392 direct ancestors of these fish back to the grandparents of the 2010 year class families. Quality control (QC) of the genotype data was performed to exclude fish with genotype call rates <0.95. Of the 941 fish with FY phenotypes and genotypes and from the 392 direct ancestors with only genotypes, were retained 875 and 391, respectively (1266 fish) that passed QC. Genotypic data from the 391 direct ancestors were used to confirm accuracy of pedigrees for the 875 phenotyped and genotyped fish used in the GWAS.

From the initial 42,488 SNPs that were polymorphic in this population, we retained those with call rates greater than 0.90, minor allele frequencies greater than 0.05, and no departures from Hardy-Weinberg equilibrium (SNPs were excluded when the difference between observed and expected genotype frequencies was >0.15). A total of 38,107 effective SNPs passed QC filtering and were used in the GWAS.

### Genome-wide association study (GWAS)

GWAS was carried out using the weighted single-step GBLUP approach (wssGWAS, Wang et al., 2012). This method combines phenotype, genotype, and pedigree data in a joint analysis and is implemented in the BLUPF90 software (Misztal et al., 2002; Aguilar et al., 2010; Wang et al., 2012). The wssGWAS avoids spurious solutions of SNPs, uses phenotypes from non-genotyped individuals included in the pedigree, and allows multi-trait analyses (Fragomeni et al., 2014; Wang et al., 2014). However, multi-trait analysis was not performed in this study due to convergence issues associated with the balance of the information. Thus, a single trait analysis was performed using the following model:

$$\begin{array}{l} y=Xb+Z_{1}a+Z_{2}w+e \end{array}$$

where *y* is the vector of the phenotypes, *b* is the vector of fixed effects including hatch year (all traits), harvest group (except BW10 and BW13), and contemporary group (only for BW10 and BW13), *a* is the vector of additive genetic effects or SNP effects, *w* is the vector of the common environment effect, and *e* is the residual error. Two co-variables were included: post-hatch age for BW10 and BW13 and harvest weight for CAR, FW, and FY. *X, Z*_1_, and *Z*_2_ are incidence matrices relating a record to fixed effects in *b* and random animal and common environment effects in *a* and *w*, respectively. The genomic (G) relationship matrix was created according to VanRaden (2008) and combined with the numerator (A) relationship matrix into a realized (H) relationship matrix to estimate additive genetic relationships among all individuals (Aguilar et al., 2010). The number of phenotypic records used in each single trait analysis is given in Table 1, and all analyses were conducted using complete pedigree data (17,706 fish) dating back to initial development of the population (8 generations). A total of 875 fish from 196 full-sib families used in the GWAS analyses had genotypic data from the SNP array and phenotypic data for all traits in Table 1.

The ssGWAS2 scenario described by Wang et al. (2014) was used to estimate genomic breeding values and iteratively estimate and weight SNP effects. Windows of 20 adjacent SNPs based on the new genetic linkage map were created using POSTGSF90 (Aguilar et al., 2014). Creation of windows of consecutive SNPs can capture the effect of the quantitative trait loci (QTL) better than a single SNP (Habier et al., 2011) due to signal concentration (Sun et al., 2011). Similar to previous reports (Dikmen et al., 2013; Fragomeni et al., 2014; Wang et al., 2014; Zhang et al., 2016), we found that the use of windows with a smaller number of SNPs resulted in a decreased signal-to-noise ratio compared to windows with 20 or more SNPs (data not shown). Therefore, exclusive windows (non-overlapping) of 20 consecutive SNPs were used in the GWAS analyses and the Manhattan plots with GWAS results were created using the R package qqman (Turner, 2014). Due to the quantitative nature of the traits used in this study, five iterations (wssGWAS) were implemented to reduce the background noise of SNP windows and differentiate the windows accounting for the largest proportion of variance. However, the differences in the results beyond 3 iterations were minimal (results not presented). Rather than using *P*-values from classical hypothesis tests to declare regions as significantly associated with the trait (Dikmen et al., 2013), here we identified genomic regions (windows) that explained the highest proportion (around 1%) of genetic variances (Wang et al., 2014).

### Putative genes network

Interpretation of the GWAS results was facilitated using network reconstruction and visualization. A network was created using SNPs in windows that explain the highest proportion of the variance for a particular trait. Selected SNPs were located in the rainbow trout genome (Berthelot et al., 2014) using the Golden Helix Genome Browser v2.1.0 (Golden Helix Inc.). When a SNP was mapped to a gene (exon or intron), the nucleotide sequence was blasted to the *Mus musculus* genome using NCBI tools to predict the orthologous genes. The mouse genome was selected because it is well annotated, is available in several bioinformatics tools, and has been used as a model organism for many years (Guenet, 2005). With the list of the genes, a network was visualized with Cytoscape (Shannon et al., 2003; Serão et al., 2013) using the BisoGenet plug-in (Martin et al., 2010; Gonzalez-Pena et al., 2016). The BisoGenet plug-in uses empirical and predicted DNA-DNA, DNA-protein, and protein-protein interactions to reconstruct and visualize likely mouse gene networks. Only gene-gene interactions were represented and only one neighbor connecting the genes was admitted. The edges denote known relationships between genes from several databases summarized in the SysBiomics repository that integrates data from NCBI, UniProt, KEGG, and GO databases using the k-nearest neighbor model. The node color denotes if the gene was identified (green) directly from the GWAS or if it is a connecting neighbor (pink) based on annotation of the mouse genome.

A functional analysis was attempted using the Database for Annotation, Visualization and Integrated Discovery (DAVID 6.7; Huang et al., 2009a,b) with the genes detected by the GWAS. However, enriched gene ontology, biological processes, molecular functions, and Kyoto Encyclopedia of Genes and Genomes (KEGG) pathways were not identified with a relevant false discovery rate. We assume that the polygenic nature of the traits was a major contributor to our inability to identify functional candidate genes near the QTL detected in the GWAS. In addition, the overall number of protein coding open reading frames we were able to find near the QTL was relatively small due to the small genome scaffolds and fragmented reference genome that is currently available for rainbow trout (Berthelot et al., 2014), which did not allow for evaluating genes on neighboring genome sequence scaffolds.

## Results and discussion

### Genetic parameter estimates

Heritability estimates (*h*^2^) for FY, FW, BW10, BW13, and CAR were moderate to high (0.31–0.62, Table 2) suggesting that these traits can be improved through selective breeding. The heritability estimates for body weight measures (BW10 and BW13) and FW were in the range of 0.19–0.50 (Elvingson and Johansson, 1993; Neira et al., 2004; Kause et al., 2007) and 0.22–0.52 (Kause et al., 2002, 2007; Powell et al., 2008; Haffray et al., 2012), respectively, as previously reported for salmonids. Similarly, the heritability estimate for FY was in the interval previously reported for salmonids (0.03–0.38), and CV was in the upper range of estimates (0.12–6.5%) (Neira et al., 2004; Kause et al., 2007; Powell et al., 2008; Haffray et al., 2012). The heritability estimate for CAR (*h*^2^ = 0.62) was higher than previous estimates of 0.36–0.53 reported in the literature (Powell et al., 2008; Haffray et al., 2012).

**Table 2:** **Genetic parameters of the traits body weight at 10 (BW10) and 13 (BW13) months post-hatching, carcass weight (CAR), fillet weight (FW), and fillet yield (FY) without (WO_GI) and with (W_GI) genomic information**.

**Parameters**	**BW10, g**		**BW13, g**		**CAR, g**		**FW, g**		**FY, %**	
	**WO_GI**	**W_GI**	**WO_GI**	**W_GI**	**WO_GI**	**W_GI**	**WO_GI**	**W_GI**	**WO_GI**	**W_GI**
$\sigma_{\text{a}}^{2}$	3399.5	4108.9	15,044	17,939	679.9	684.5	363.0	412.8	1.8	1.9
$\sigma_{\text{w}}^{2}$	1427.4	1346.1	6344.1	6316.7	46.0	19.7	10.6	7.6	0.6	0.3
$\sigma_{\text{e}}^{2}$	6031.5	5659.4	24,979	23,482	362.7	355.5	533.8	510.8	3.0	3.1
$\sigma_{\text{p}}^{2}$	10,858.4	11,114.4	46,367.1	47,737.7	1088.6	1059.6	907.4	931.2	5.4	5.4
h^2^	0.31	0.37	0.32	0.38	0.62	0.65	0.40	0.44	0.34	0.36
Acc	0.66	0.70	0.65	0.69	0.28	0.55	0.25	0.50	0.13	0.55
r(EBV,GEBV)		0.99		0.99		0.76		0.79		0.72

*$\sigma_{a}^{\text{2}}$, additive variance; $\sigma_{w}^{\text{2}}$, common environment variance; $\sigma_{e}^{\text{2}}$, residual variance; $\sigma_{p}^{\text{2}}$, phenotypic variance; h^2^, heritability (additive variance/phenotypic variance); Acc, average accuracy; r(EBV,GEBV), correlation between estimated breeding values (EBV) and genomic estimated breeding values (GEBV)*.

The correlations between estimated breeding values (EBVs) and genomic breeding values (GEBVs) were near unity (0.99) for traits measured directly on breeding candidates (BW10 and BW13) and smaller (0.72–0.79) for lethally-measured traits (CAR, FW, and FY; Table 2). In the absence of progeny performance data or a correlated trait that can be measured directly on breeding candidates, traditional BLUP-based EBVs for CAR, FW, and FY are necessarily identical among non-phenotyped siblings, whereas the use of genomic information (GI) in GBLUP enables fish-specific GEBVs despite the absence of phenotypic data for the fish, its progeny, or for a correlated trait. Thus, the smaller correlations between EBVs and GEBVs for lethally-measured traits was expected because the correlation is between family-specific and fish-specific estimates of genetic merit, respectively. When GI was added to the aforementioned analysis, a slight increase in heritabilities was observed for all traits (Table 2). However, the accuracies of GEBVs were increased by ~100% for CAR and FW (from 0.28 and 0.25 to 0.55 and 0.50, respectively) and by ~420% for FY (from 0.13 to 0.55) compared traditional pedigree-based EBVs. For lethally-measured traits like FW, FY, and CAR, traditional selective breeding programs rely on sib-testing with limited reliability (Odegård et al., 2014). Therefore, methods that increase accuracy of predictions and expedite genetic progress by exploiting within-family genetic variation for economically-important traits in aquaculture species are important for continued development of the aquaculture industry (Yáñez et al., 2015). Models that include GI from numerous SNP markers in addition to the phenotypic and pedigree information without previous knowledge of the underlying QTL outperformed models without GI (Nielsen et al., 2009; Odegård et al., 2014). This improved performance of GI models is expected based on the definition of the accuracy as a function of heritability and amount of information used (Chen et al., 2011). Examples of traits for which inclusion of GI resulted in an increase in accuracy include growth in broiler chickens (Wang et al., 2014); lice resistance and fillet color in Atlantic salmon (Odegård et al., 2014); and weight and length traits in Atlantic salmon (Tsai et al., 2015).

### GWAS

**Figure 1:**
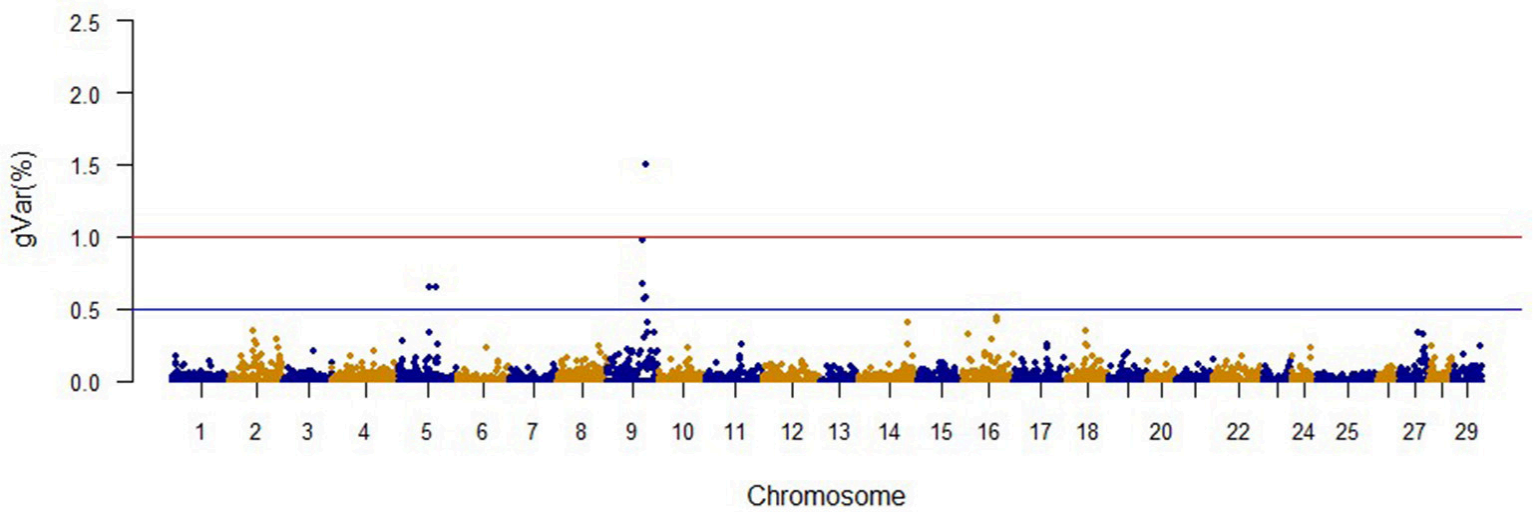
**The proportion of genetic variance explained by 20-SNP regions for fillet yield**.

**Figure 2:**
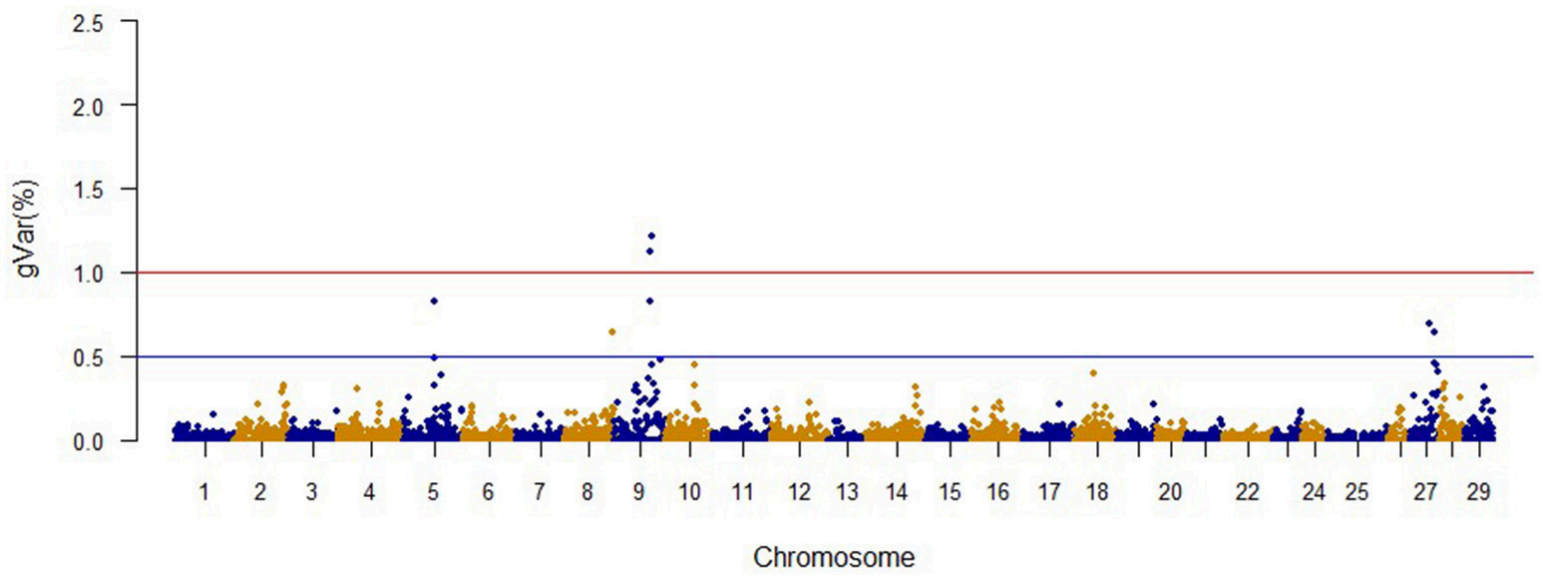
**The proportion of genetic variance explained by 20-SNP regions for fillet weight**.

**Figure 3:**
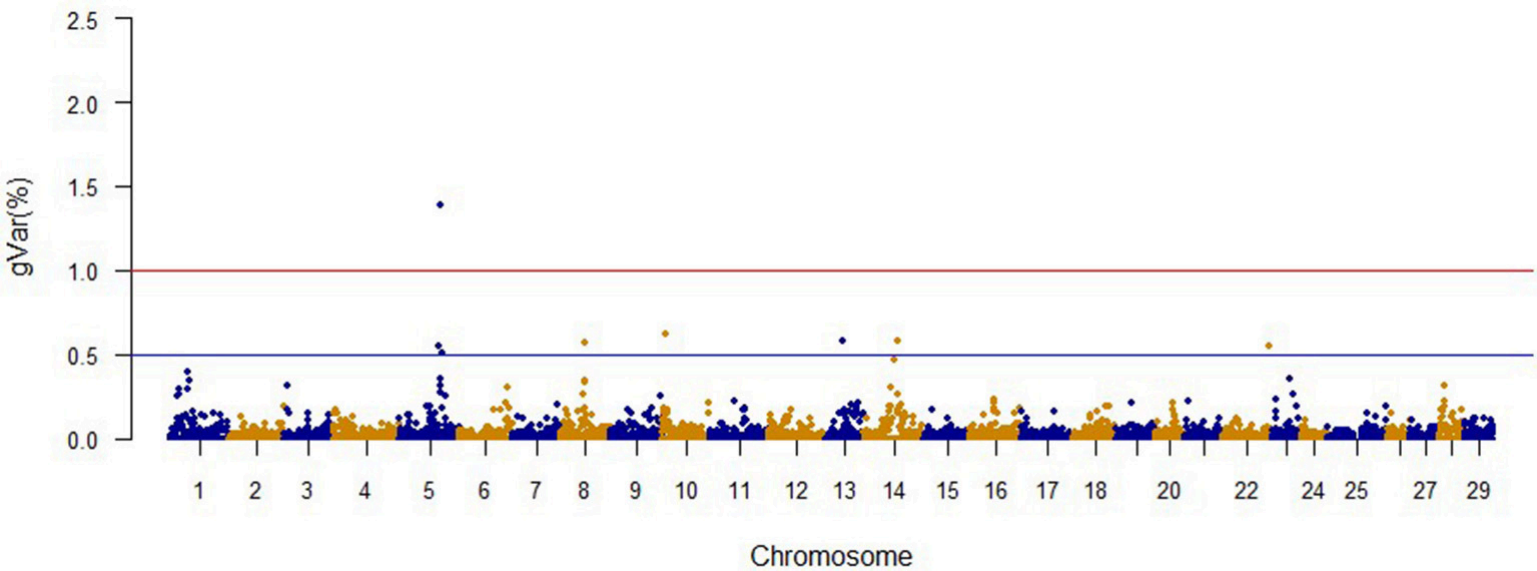
**The proportion of genetic variance explained by 20-SNP regions for 10-month body weight**.

**Figure 4:**
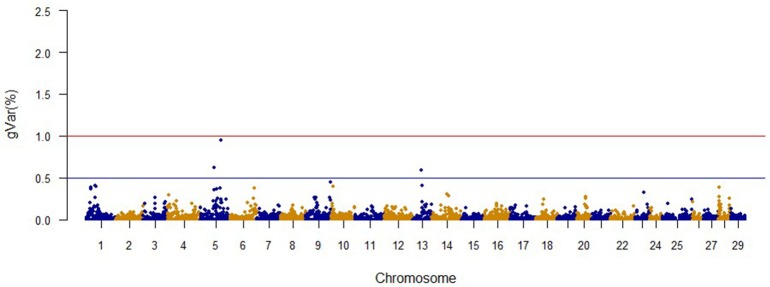
**The proportion of genetic variance explained by 20-SNP regions for 13-month body weight**.

**Figure 5:**
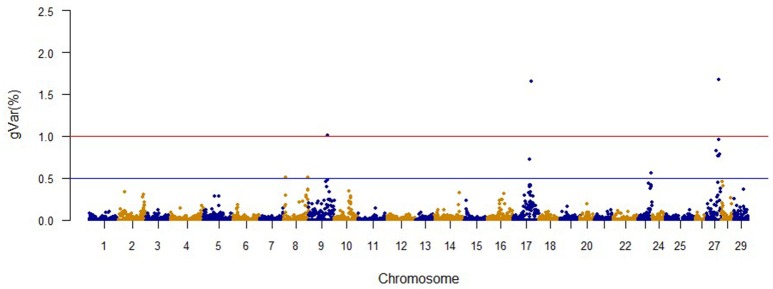
**The proportion of genetic variance explained by 20-SNP regions for carcass weight**.

The Manhattan plots from GWAS results at iteration 5 for FY, FW, BW10, BW13, and CAR are shown in Figures 1–5, respectively. In total, 1906 non-overlapping, non-repetitive windows of 20 successive SNPs were used. Of these windows, two windows located on chromosome Omy9 explained 1.5 and 1.0% of the genetic variance for FY. The same windows explained 1.2 and 1.1% of the genetic variance for FW. Only one window, located on Omy5, was responsible for 1.38 and 0.95% of the genetic variance for BW10 and BW13, respectively. Three windows located on Omy27, Omy17, and Omy9 were responsible for 1.7, 1.7, and 1.0%, of the genetic variance in CAR, respectively.

No major QTL was detected for FY, suggesting that this trait has a polygenic architecture affected by multiple loci with small effects in this rainbow trout population. The SNP markers from the two windows that explained at least 1.0% of the proportion of variance for FY and harboring or neighboring genes from the same genome scaffold (Berthelot et al., 2014) are listed in Table 3. An extended list of all the SNPs markers is provided in Table S2. Only 13 of the 40 SNPs identified by the wssGWAS on Omy9 explained a proportion of the genetic variance equal to or greater than 0.1%. Of them, nine were located within a gene (exon or intron) and the other four had known neighboring genes in the same genome scaffold. Four of those 13 SNPs were located in scaffold_516, of which one SNP was mapped to exon 6 of Properdin, another to exon 3 of Transmembrane protein 201-like, and two SNPs were mapped in the intron regions of Calsyntenin-1-like isoform x2 (Table 3). Briefly, Properdin is one of the proteins that participates in the complement system, which also may interfere with fatty acid uptake and esterification in adipocytes (Gauvreau et al., 2012). Transmembrane protein 201-like is a component of transmembrane actin-associated nuclear lines with a role in centrosome orientation and nuclear movement prior to cell migration (Borrego-Pinto et al., 2012). Calsyntenin-1 is associated with kinesin-1-mediated transport of vesicles and tubulovesicular organelles (Konecna et al., 2006), appears to participate in intracellular transport and endosomal trafficking, and is necessary for the formation of peripheral sensory axons (Ponomareva et al., 2014). However, the biological functions of calsyntenins are not well understood yet (Ortiz-Medina et al., 2015).

**Table 3:** **The SNP markers that explained the largest proportion of variance for fillet yield using 20-SNP windows**.

**Marker**	**Chr**	**Position (cM)**	**Alleles**	**VE (%)**	**Scaffold**	**Scaffold position**	**Scaffold size**	**Loc**	**Description**
**WINDOW 1 TOTAL PROPORTION 1.5%**									
AX-89976492	9	125.19	T/C	0.13	Scaffold_8612	10,780	26,627	Intron	Beta-catenin-interacting protein 1 isoform x1
AX-89970327	9	125.19	A/C	0.11	Scaffold_516	399,414	728,099	Intron	Calsyntenin-1-like isoform x2
AX-89940136	9	125.19	A/G	0.12	Scaffold_516	407,663	728,099	Intron	Calsyntenin-1-like isoform x2
AX-89936139	9	125.67	A/C	0.13				Near^*^	None
AX-89944669	9	125.67	G/T	0.13	Scaffold_32707	921	3946	Exon 1	Nudix hydrolase chloroplastic-like
AX-89940514	9	125.67	T/C	0.12	Scaffold_10308	15,430	21,663	Near^*^	None
AX-89937961	9	125.67	G/T	0.13	Scaffold_516	586,177	728,099	Exon 6	Properdin
AX-89951506	9	125.67	G/A	0.12	Scaffold_516	555,991	728,099	Exon 3	Transmembrane protein 201-like
AX-89938525	9	125.86	G/A	0.10	Scaffold_52	1,795,215	2,128,772	Intron	Atpase family aaa domain-containing protein 3-like
AX-89957923	9	126.04	A/G	0.13	Scaffold_19674	6079	8261	Near^*^	None
**WINDOW 2 TOTAL PROPORTION 1.0%**									
AX-89951447	9	117.12	G/A	0.10	Scaffold_43535	864	3113	Near^*^	None
AX-89940159	9	117.12	T/C	0.11	Scaffold_347	148,757	935,129	Intron	Serine threonine-protein kinase sbk1-like
AX-89924961	9	117.91	C/T	0.10	Scaffold_347	414,782	935,129	Exon 6	Phd finger protein 20-like isoform x3

*Chr, chromosome; VE, percentage of the genetic variance explained by the SNP; Loc, location in the scaffold with three possibilities: intron, exon, or near an exon*.

The same two windows found in the FY GWAS were also associated with FW. However, from the 40 SNPs markers identified by the wssGWAS, only 12 explained a proportion equal to or greater than 0.1% and of them, nine were in a gene (exon or intron, Table 4). An extended list of all the SNP markers is provided in Table S3. Similar to FY, no major QTL was detected for FW which supports the polygenic architecture of FW. Three of the 12 SNPs were also located in scaffold_516 within the Calsyntenin-1-like isoform x2, Properdin and Transmembrane protein 201-like genes (Table 4). Three additional SNPs were located in scaffold_347 in the Src-like-adapter 2; Serine/ threonine-protein kinase sbk1-like; and Phd finger protein 20-like isoform x3 genes. Briefly, Src-like-adapter 2 is an adaptor protein that regulates T and B cell maturation and development, and it is a critical component regulating signal transduction in immune and malignant cells (Sosinowski et al., 2001; Dragone et al., 2006; Kazi et al., 2015). Additionally, differentially-expressed transcripts in response to handling and confinement stress in rainbow trout were mapped to a serine/threonine-protein kinase, SBK1, homologous in zebrafish that participates in signal transduction pathways related to brain development (Chou et al., 2006; Liu et al., 2015a).

**Table 4:** **The SNP markers that explained the largest proportion of variance for fillet weight using 20-SNP windows**.

**Marker**	**Chr**	**Position (cM)**	**Alleles**	**VE (%)**	**Scaffold**	**Scaffold position**	**Scaffold size**	**Loc**	**Description**
**WINDOW 1 TOTAL PROPORTION 1.2%**									
AX-89976492	9	125.19	T/C	0.12	Scaffold_8612	10,780	26,627	Intron	Beta-catenin-interacting protein 1 isoform x1
AX-89970327	9	125.19	A/C	0.10	Scaffold_516	399,414	728,099	Intron	Calsyntenin-1-like isoform x2
AX-89936139	9	125.67	A/C	0.11				Near^*^	None
AX-89944669	9	125.67	G/T	0.11	Scaffold_32707	921	3946	Exon 1	Nudix hydrolase chloroplastic-like
AX-89940514	9	125.67	T/C	0.10	Scaffold_10308	15,430	21,663	Near^*^	None
AX-89937961	9	125.67	G/T	0.11	Scaffold_516	586,177	728,099	Exon 6	Properdin
AX-89951506	9	125.67	G/A	0.11	Scaffold_516	555,991	728,099	Exon 3	Transmembrane protein 201-like
**WINDOW 2 TOTAL PROPORTION 1.1%**									
AX-89953042	9	116.09	G/A	0.10	Scaffold_1609	166,168	200,099	Exon 4	Kelch domain-containing protein 8b
AX-89951447	9	117.12	G/A	0.13	Scaffold_43535	864	3113	Near^*^	None
AX-89975284	9	117.12	A/C	0.11	Scaffold_347	348,224	935,129	Exon 7	Src-like-adapter 2
AX-89940159	9	117.12	T/C	0.12	Scaffold_347	148,757	935,129	Intron	Serine threonine-protein kinase sbk1-like
AX-89924961	9	117.91	C/T	0.13	Scaffold_347	414,782	935,129	Exon 6	Phd finger protein 20-like isoform x3

*Chr, chromosome; VE, percentage of the genetic variance explained by the SNP; Loc, location in the scaffold with three possibilities: intron, exon, or near an exon*.

Genome regions associated with growth have been detected on most of the 29 chromosomes in Atlantic salmon (Baranski et al., 2010; Gutierrez et al., 2012, 2015; Tsai et al., 2015) and in rainbow trout (O'Malley et al., 2003; Perry et al., 2005; Wringe et al., 2010). The heterogeneity in the results makes it hard to compare our results to previous studies. Many factors contribute to this observed heterogeneity between studies including: (1) the highly polygenic architecture of growth and growth-related traits; (2) differences in marker segregation that may be affected by the strain genetic background, and different types and densities of markers used in each study; (3) different algorithms used in the QTL detection analyses; (4) large variation in sample size (Baranski et al., 2010; Wringe et al., 2010; Tsai et al., 2015), and (5) possible false positives. None of the 20 SNPs identified by the wssGWAS for BW10 and BW13 on chromosome Omy5 (Figures 3, 4) were able to surpass the threshold of 0.1%. The complete list of the SNPs is provided in Tables S4, S5 for BW10 and BW13, respectively. Our findings of the polygenic architecture of growth traits in fish is consistent with previous reports in the literature (Devlin et al., 2009; Dai et al., 2015; Tsai et al., 2015), and, congruent with our GWAS results, several markers were associated with weight in a GWAS for Atlantic salmon, but the proportion of variance explained by each marker was less than 0.1% (Tsai et al., 2015).

Lastly, SNP markers that explained more than 0.1% of the proportion of genetic variance for CAR on Omy27, 17, and 9, and harboring or neighboring genes from the same genome scaffold (Berthelot et al., 2014) are listed in Table 5. No major QTL was detected for this trait. An extended list of all the SNP markers is provided in Table S6. From the 60 SNP markers identified by wssGWAS, only 18 explained a proportion equal to or greater than 0.1%, of which 10 were in a gene (exon or intron). Four of the 18 SNPs were located in scaffold_173; one was in the exon of ubiquitin-conjugating enzyme e2 variant 1, and three were near this gene and near the histone h2b 1 2-like gene. One of the SNPs was mapped to the Calsyntenin-1-like isoform x2 intron region in scaffold_516 that also affected FY and FW.

**Table 5:** **The SNP markers that explained the largest proportion of variance for carcass weight using 20-SNP windows**.

**Marker**	**Chr**	**Position (cM)**	**Alleles**	**VE (%)**	**Scaffold**	**Scaffold position**	**Scaffold size**	**Loc**	**Description**
**WINDOW 1 TOTAL PROPORTION 1.7%**									
AX-89952551	27	75.09	A/G	0.12	Scaffold_1006	143,453	383,627	Near	nitric oxide inducible/serine threonine-protein kinase nlk
AX-89954149	27	75.09	C/A	0.12	Scaffold_147	921,922	1,497,438	Near	atp-sensitive inward rectifier potassium channel 1-like/cmp-n-acetylneuraminate-beta-galactosamide-alpha-sialyltransferase 4-like isoform x1
AX-89938133	27	75.09	A/G	0.13	Scaffold_1006	46,265	383,627	Exon3	nitric oxide inducible
AX-89948564	27	74.78	G/A	0.12	Scaffold_8798	5532	26,005	Near	Undetermined/None
AX-89974542	27	74.58	G/T	0.11	Scaffold_842	38,757	463,739	Intron	kinase suppressor of ras 1-like isoform x2
AX-89926230	27	74.58	A/G	0.12	Scaffold_1952	116,665	147,230	Near	neurofibromin isoform x2/oligodendrocyte-myelin glyco
AX-89938965	27	74.43	G/T	0.12	Scaffold_842	38,933	463,739	Intron	kinase suppressor of ras 1-like isoform x2
AX-89928353	27	73.96	G/A	0.10	Scaffold_1675	177,158	191,261	Intron	vascular endothelial zinc finger 1-like isoform x2
AX-89968747	27	73.96	A/G	0.11	Scaffold_3611	60,104	62,580	Intron	unconventional myosin-xviiia-like isoform x1
AX-89947091	27	73.96	na	0.10				na	Na
AX-89942611	27	73.42	C/A	0.16	Scaffold_3980	33,385	57,002	Intron	unconventional myosin-xviiia-like isoform x2
**WINDOW 2 TOTAL PROPORTION 1.7%**									
AX-89973675	17	115.87	G/T	0.10	Scaffold_26752	3396	4948	Intron	spectrin beta non-erythrocytic 1-like
AX-89969602	17	114.85	C/T	0.10	Scaffold_24	197,378	2,579,057	Intron	calpain-2 catalytic subunit-like
AX-89935000	17	114.02	A/G	0.17	Scaffold_173	119,365	1,392,108	Near	histone h2b 1 2-like/ubiquitin-conjugating enzyme e2 variant 1
AX-89918454	17	114.02	C/A	0.12	Scaffold_173	123,912	1,392,108	Near	histone h2b 1 2-like/ubiquitin-conjugating enzyme e2 variant 1
AX-89923840	17	113.51	C/T	0.18	Scaffold_173	195,297	1,392,108	Exon4	ubiquitin-conjugating enzyme e2 variant 1
AX-89925576	17	113.51	A/G	0.18	Scaffold_173	160,398	1,392,108	Near	histone h2b 1 2-like/ubiquitin-conjugating enzyme e2 variant 1
**WINDOW 3 TOTAL PROPORTION 1.0%**									
AX-89940136	9	125.19	A/G	0.10	Scaffold_516	407,663	728,099	Intron	Calsyntenin-1-like isoform x2

*Chr, chromosome; VE, percentage of the genetic variance explained by the SNP; Loc, location in the scaffold with three possibilities: intron, exon, or near an exon*.

### Putative genes

**Figure 6:**
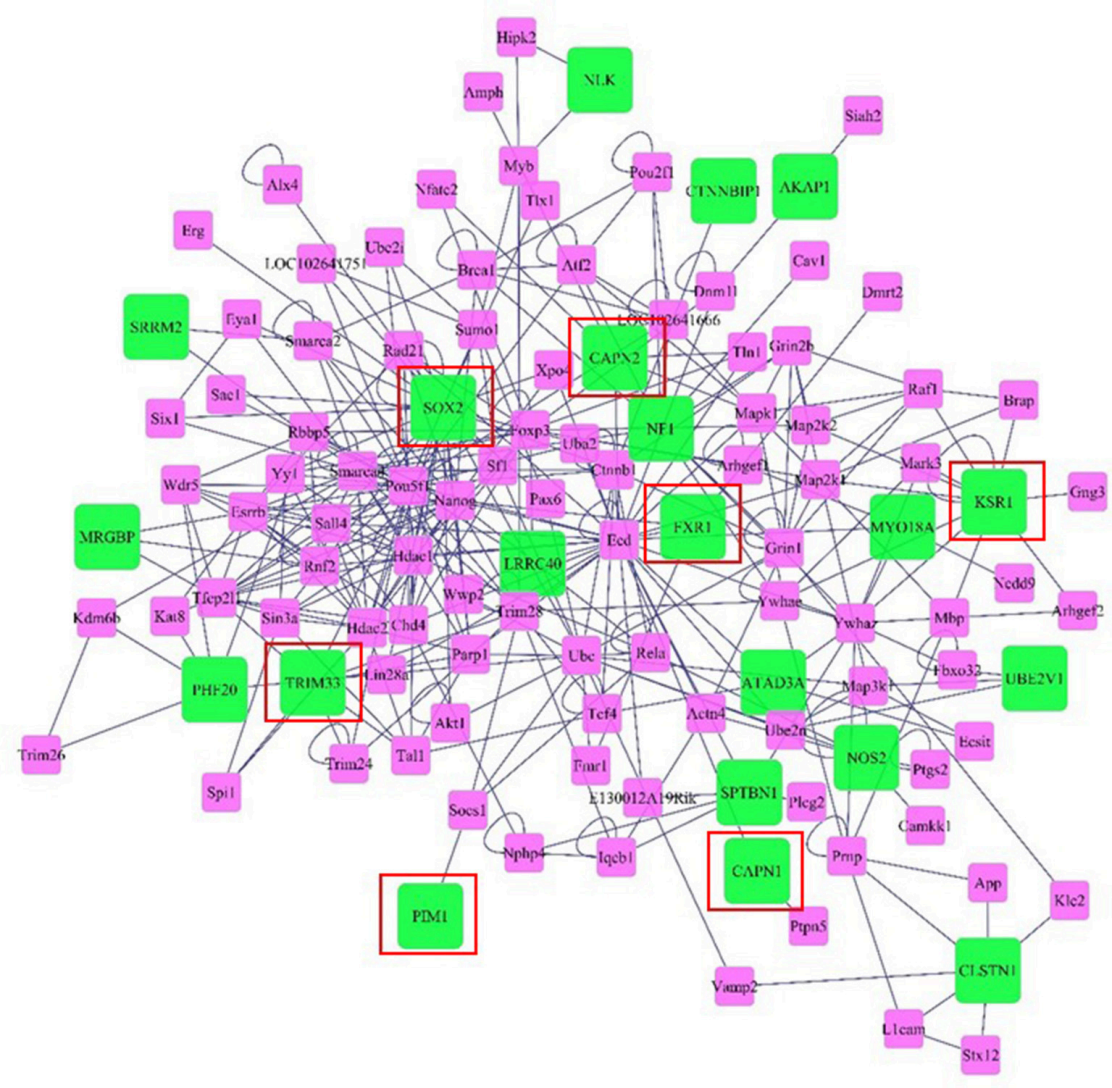
**Network of genes and neighboring genes in the same scaffold as the SNPs identified in the wssGWAS in windows responsible for ~1.0% or more of the total genetic variance of the analyzed traits**. Green nodes denote genes and near genes detected by the wssGWAS analysis and pink nodes denote connecting neighbors. Edges denote known relationships between genes in the SysBiomics repository. Framed genes (red square) are discussed in the manuscript.

Networks of genes offer insight to the molecular relationships among genes. The network was visualized considering genes and neighboring genes located in the same genome scaffold as the SNPs we identified in the wssGWAS in 20-SNP windows responsible for ~1.0% or more of the total genetic variance for the analyzed traits. Genes included in the network are described in Tables S2–S6. The network includes 115 gene nodes, of which 21 nodes were genes detected by the wssGWAS analysis (green nodes) while the rest were connecting neighbors (pink nodes, Figure 6). In this network, SRY (sex determining region Y)-box 2 (Sox2), Kinase suppressor of ras 1 (Ksr1), Tripartite motif-containing 33 (Trim33), and Nitric oxide synthase 2 inducible (Nos2) were well-connected gene nodes linking to 29, 11, 7, and 6 other gene nodes, respectively.

A number of genes detected by wssGWAS analysis within windows responsible for 1.0% or greater of the trait variance have been identified in mammalian systems as significant for muscle development, particularly with respect to the maintenance of hyperplastic capacity. Rainbow trout exhibit indeterminate growth potential as they have the ability increase both body length and muscle mass throughout adulthood (Johnston et al., 2011). A physiological mechanism unique to indeterminate growers is the ability to maintain a population of myogenic precursor cells with proliferative capacity that then differentiate into myotubes or contribute to nuclear accretion (Mommsen, 2001; Froehlich et al., 2013). Therefore, the continued ability for hyperplasia and hypertrophy in skeletal muscle contributes to the indeterminate growth phenotype (Johnston et al., 2011). In this regard, SNPs affecting genes related to myogenic and cell proliferation mechanisms are expected to affect growth and fillet yield.

One gene detected by wssGWAS analysis that was the most connected node was Sox2. The Sox2 gene plays a critical role in maintenance and proliferation of pluripotent and neural progenitor stem cells (Takahashi and Yamanaka, 2006; Zhang and Cui, 2014) through its interaction with transforming Growth Factor β (TGFb) signaling (Gaarenstroom and Hill, 2014), although little is known about the role of Sox2 in muscle. Albeit, TGFb ligands like myostatin inhibit muscle growth (Lee et al., 2005; Phelps et al., 2013), partially through reductions in myogenic precursor cell proliferation (Garikipati and Rodgers, 2012; Seiliez et al., 2012). Trim33 is another gene detected by wssGWAS analysis that is up-regulated during muscle regeneration in mice and appears to play a role in myoblast proliferation (Mohassel et al., 2015). Therefore, similar to Sox2, Trim33 may also contribute to maintaining a proliferating population of myogenic precursor cells throughout development in the rainbow trout. Trim33 also inhibits Smad4 (Xi et al., 2011), a transcription factor activated by TGFb signaling that inhibits muscle regeneration and maintenance of myogenesis with age (Lee et al., 2005).

A third gene identified by wssGWAS analysis was Fragile X mental retardation gene 1 (Fxr1), an autosomal gene that is highly expressed in muscle (Blonden et al., 2005). Depletion of this gene during early development of the zebrafish leads to cardiomyopathy and muscular dystrophy (Van't Padje et al., 2009). In cultured muscle cells, depletion of Fxr1 reduced myoblast abundance, suggesting an evolutionarily-conserved role for Fxr1 protein in myogenesis (Davidovic et al., 2013). A fourth gene detected by wssGWAS analysis that may affect myogenic capacity was Ksr1. This gene is a scaffold protein for the Raf/MEK/ERK kinase cascade (Ory et al., 2003). Although, a role of for Ksr1 in muscle has not been demonstrated, activation of the Raf/Mek/ERK kinase cascade promotes proliferation of myogenic cells (Knight and Kothary, 2011). A final gene that may affect myogenesis was Proviral integration site 1 (Pim1), a gene that, when overexpressed in cardiomyocytes, causes increases in regenerative capacity and increases the pool of progenitor cells (Del Re and Sadoshima, 2012), although it is unknown if this gene has significance in skeletal muscle. However, expression of Pim1 has been positively correlated with intramuscular fat content in steers (Sadkowski et al., 2014).

Another pair of genes that were detected by wssGWAS and are known to encode proteins with functional importance to muscle physiology are the cysteine proteases Calpain-1 (Capn1) and Calpain-2 (Capn2). Calpains are calcium activated cysteine proteases regulated by myogenic factors like myoD and myogenin (Dedieu et al., 2003). Calpains have a relevant role in signal transduction (Glading et al., 2001; Sato and Kawashima, 2001), cell-cycle regulation, apoptosis (Atencio et al., 2000; Patel and Lane, 2000), cell spreading and migration (Dourdin et al., 1997; Huttenlocher et al., 1997; Potter et al., 1998), and myogenesis (Barnoy et al., 2000). Calpains are also involved with myofibrillar protein disassembly and degradation, contributing to loss of the Z disk (Busch et al., 1972). Increased calpain activity in muscle occurs during periods of muscle reorganization and restructuring (Dedieu et al., 2004), such as during weight loss and rapid growth (Salem et al., 2005; Johnston et al., 2011; Salmerón et al., 2015), therefore it is feasible that SNPs affecting calpain-related proteolysis contributes to differences in muscle growth capacity. Calpain-induced protein degradation is also associated with post-mortem proteolysis so genetic variation in the calpain system may result in differences in fillet quality (Koohmaraie, 1992; Delbarre-Ladrat et al., 2006).

## Conclusions

The use of genomic information from the whole-genome association analyses in this study increased the heritability and accuracy of estimated breeding values for FY, suggesting that genomic selection will be suitable for exploiting within-family genetic variation and thus obtaining faster progress in selective breeding for this trait. Only few windows were able to explain more than 1% of the genetic variance of FY, FW, BW10, BW13, and CAR, thus corroborating the polygenic nature of these traits. Network visualization of the putative genes implicated in the GWAS analyses indicated that differences in the ability of the fish to maintain a proliferative and renewable population of myogenic precursor cells might be affecting the observed phenotypic and genetic variance in growth rate and fillet yield in rainbow trout.

## Author contributions

YP, TL conceived and planned the study; PK, TL were responsible for phenotyping; GG, YP were responsible for high-density SNP genotyping and quality control of genotypic data; MB, TM, GG, and YP developed the linkage map; DG, RV were responsible for planning and conducting the GWAS analyses; BC, DG developed and interpreted gene networks; and DG drafted the manuscript. All authors read and approved the final manuscript.

### Conflict of interest statement

The authors declare that the research was conducted in the absence of any commercial or financial relationships that could be construed as a potential conflict of interest.

## Supplementary material

Table S1 **The rainbow trout genetic linkage map**.

Table S2 **The 40 SNP markers from the two windows that explained the largest proportion of variance for FY and harboring or neighboring genes from the same genome scaffold (Berthelot et al., 2014)**.

Table S3 **The 40 SNP markers from the two windows that explained the largest proportion of variance for FW and harboring or neighboring genes from the same genome scaffold (Berthelot et al., 2014)**.

Table S4 **The 20 SNP markers from the one window that explained the largest proportion of variance for BW10 and harboring or neighboring genes from the same genome scaffold (Berthelot et al., 2014)**.

Table S5 **The 20 SNP markers from the one window that explained the largest proportion of variance for BW13 and harboring or neighboring genes from the same genome scaffold (Berthelot et al., 2014)**.

Table S6 **The 60 SNP markers from the three windows that explained the largest proportion of variance for CAR and harboring or neighboring genes from the same genome scaffold (Berthelot et al., 2014)**.

## References

[B1] Aguilar I. Misztal I. Johnson D. L. Legarra A. Tsuruta S. Lawlor T. J. (2010). Hot topic: a unified approach to utilize phenotypic, full pedigree, and genomic information for genetic evaluation of Holstein final score. J. Dairy Sci. 93, 743–752. 10.3168/jds.2009-2730 20105546

[B2] Aguilar I. Misztal I. Tsuruta S. Legarra A. Wang H. (2014). PREGSF90 – POSTGSF90: computational tools for the implementation of single-step genomic selection and genome-wide association with ungenotyped individuals in BLUPF90 programs, in Proceedings, 10th World Congress of Genetics Applied to Livestock Production (Vancouver, BC).

[B3] Atencio I. A. Ramachandra M. Shabram P. Demers G. W. (2000). Calpain inhibitor 1 activates p53-dependent apoptosis in tumor cell lines. Cell Growth Differ. 11, 247–253. 10845425

[B4] Baranski M. Moen T. Våge D. I. (2010). Mapping of quantitative trait loci for flesh colour and growth traits in Atlantic salmon (Salmo salar). Genet. Sel. Evol. 42, 17. 10.1186/1297-9686-42-17 20525320 PMC2900243

[B5] Barnoy S. Supino-Rosin L. Kosower N. S. (2000). Regulation of calpain and calpastatin in differentiating myoblasts: mRNA levels, protein synthesis and stability. Biochem. J. 351 (Pt 2), 413–20. 10.1042/bj3510413 11023827 PMC1221377

[B6] Berthelot C. Brunet F. Chalopin D. Juanchich A. Bernard M. Noël B.. (2014). The rainbow trout genome provides novel insights into evolution after whole-genome duplication in vertebrates. Nat. Commun. 5:3657. 10.1038/ncomms4657 24755649 PMC4071752

[B7] Blonden L. van't Padje S. Severijnen L.-A. Destree O. Oostra B. A. Willemsen R. (2005). Two members of the Fxr gene family, Fmr1 and Fxr1, are differentially expressed in Xenopus tropicalis. Int. J. Dev. Biol. 49, 437–441. 10.1387/ijdb.051974lb 15968590

[B8] Borrego-Pinto J. Jegou T. Osorio D. S. Auradé F. Gorjánácz M. Koch B.. (2012). Samp1 is a component of TAN lines and is required for nuclear movement. J. Cell Sci. 125, 1099–1105. 10.1242/jcs.087049 22349700

[B9] Bugeon J. Lefevre F. Cardinal M. Uyanik A. Davenel A. Haffray P. (2010). FLESH QUALITY IN LARGE RAINBOW TROUT WITH HIGH OR LOW FILLET YIELD. J. Muscle Foods 21, 702–721. 10.1111/j.1745-4573.2010.00214.x26472703

[B10] Busch W. A. Stromer M. H. Goll D. E. Suzuki A. (1972). Ca 2+ -specific removal of Z lines from rabbit skeletal muscle. J. Cell Biol. 52, 367–381. 10.1083/jcb.52.2.367 PMC2108631 4621650

[B11] Chen C. Y. Misztal I. Aguilar I. Tsuruta S. Meuwissen T. H. E. Aggrey S. E.. (2011). Genome-wide marker-assisted selection combining all pedigree phenotypic information with genotypic data in one step: an example using broiler chickens. J. Anim. Sci. 89, 23–28. 10.2527/jas.2010-3071 20889689

[B12] Chou C.-M. Chen Y.-C. Lee M.-T. Chen G.-D. Lu I.-C. Chen S.-T.. (2006). Expression and characterization of a brain-specific protein kinase BSK146 from zebrafish. Biochem. Biophys. Res. Commun. 340, 767–775. 10.1016/j.bbrc.2005.12.090 16403448

[B13] Christensen O. F. Lund M. S. (2010). Genomic prediction when some animals are not genotyped. Genet. Sel. Evol. 42:2. 10.1186/1297-9686-42-2 20105297 PMC2834608

[B14] Correa K. Lhorente J. P. López M. E. Bassini L. Naswa S. Deeb N.. (2015). Genome-wide association analysis reveals loci associated with resistance against *Piscirickettsia salmonis* in two Atlantic salmon (*Salmo salar* L.) chromosomes. BMC Genomics 16:854. 10.1186/s12864-015-2038-7 26499328 PMC4619534

[B15] Dai X. Zhang W. Zhuo Z. He J. Yin Z. (2015). Neuroendocrine regulation of somatic growth in fishes. Sci. China. Life Sci. 58, 137–147. 10.1007/s11427-015-4805-8 25655896

[B16] Davidovic L. Durand N. Khalfallah O. Tabet R. Barbry P. Mari B.. (2013). A novel role for the RNA-binding protein FXR1P in myoblasts cell-cycle progression by modulating p21/Cdkn1a/Cip1/Waf1 mRNA stability. PLoS Genet. 9:e1003367. 10.1371/journal.pgen.1003367 23555284 PMC3605292

[B17] Dedieu S. Mazères G. Dourdin N. Cottin P. Brustis J.-J. (2003). Transactivation of capn2 by myogenic regulatory factors during myogenesis. J. Mol. Biol. 326, 453–465. 10.1016/S0022-2836(02)01310-4 12559913

[B18] Dedieu S. Poussard S. Mazères G. Grise F. Dargelos E. Cottin P.. (2004). Myoblast migration is regulated by calpain through its involvement in cell attachment and cytoskeletal organization. Exp. Cell Res. 292, 187–200. 10.1016/j.yexcr.2003.08.014 14720518

[B19] Dekkers J. C. M. (2012). Application of genomics tools to animal breeding. Curr. Genomics 13, 207–212. 10.2174/138920212800543057 23115522 PMC3382275

[B20] Delbarre-Ladrat C. Chéret R. Taylor R. Verrez-Bagnis V. (2006). Trends in postmortem aging in fish: understanding of proteolysis and disorganization of the myofibrillar structure. Crit. Rev. Food Sci. Nutr. 46, 409–421. 10.1080/10408390591000929 16891212

[B21] Del Re D. P. Sadoshima J. (2012). Enhancing the potential of cardiac progenitor cells: pushing forward with Pim-1. Circ. Res. 110, 1154–1156. 10.1161/CIRCRESAHA.112.269183 22539751 PMC3350802

[B22] Devlin R. H. Sakhrani D. Tymchuk W. E. Rise M. L. Goh B. (2009). Domestication and growth hormone transgenesis cause similar changes in gene expression in coho salmon (Oncorhynchus kisutch). Proc. Natl. Acad. Sci. U.S.A. 106, 3047–3052. 10.1073/pnas.0809798106 19223591 PMC2651260

[B23] Dikmen S. Cole J. B. Null D. J. Hansen P. J. (2013). Genome-wide association mapping for identification of quantitative trait loci for rectal temperature during heat stress in Holstein cattle. PLoS ONE 8:e69202. 10.1371/journal.pone.0069202 23935954 PMC3720646

[B24] Dourdin N. Brustis J. J. Balcerzak D. Elamrani N. Poussard S. Cottin P.. (1997). Myoblast fusion requires fibronectin degradation by exteriorized m-calpain. Exp. Cell Res. 235, 385–394. 10.1006/excr.1997.3684 9299163

[B25] Dragone L. L. Myers M. D. White C. Sosinowski T. Weiss A. (2006). SRC-like adaptor protein regulates B cell development and function. J. Immunol. 176, 335–345. 10.4049/jimmunol.176.1.335 16365426

[B26] Elvingson P. Johansson K. (1993). Genetic and environmental components of variation in body traits of rainbow trout (*Oncorhynchus mykiss*) in relation to age. Aquaculture 118, 191–204. 10.1016/0044-8486(93)90456-9

[B27] Fragomeni B. D. O. Misztal I. Lourenco D. L. Aguilar I. Okimoto R. Muir W. M. (2014). Changes in variance explained by top SNP windows over generations for three traits in broiler chicken. Front. Genet. 5:332. 10.3389/fgene.2014.00332 25324857 PMC4181244

[B28] Froehlich J. M. Fowler Z. G. Galt N. J. Smith D. L. Biga P. R. (2013). Sarcopenia and piscines: the case for indeterminate-growing fish as unique genetic model organisms in aging and longevity research. Front. Genet. 4:159. 10.3389/fgene.2013.00159 23967015 PMC3743216

[B29] Gaarenstroom T. Hill C. S. (2014). TGF-β signaling to chromatin: how Smads regulate transcription during self-renewal and differentiation. Semin. Cell Dev. Biol. 32, 107–118. 10.1016/j.semcdb.2014.01.009 24503509

[B30] Garikipati D. K. Rodgers B. D. (2012). Myostatin inhibits myosatellite cell proliferation and consequently activates differentiation: evidence for endocrine-regulated transcript processing. J. Endocrinol. 215, 177–187. 10.1530/JOE-12-0260 22872758

[B31] Gauvreau D. Roy C. Tom F.-Q. Lu H. Miegueu P. Richard D.. (2012). A new effector of lipid metabolism: complement factor properdin. Mol. Immunol. 51, 73–81. 10.1016/j.molimm.2012.02.110 22387270

[B32] Geng X. Sha J. Liu S. Bao L. Zhang J. Wang R.. (2015). A genome-wide association study in catfish reveals the presence of functional hubs of related genes within QTLs for columnaris disease resistance. BMC Genomics 16:196. 10.1186/s12864-015-1409-4 25888203 PMC4372039

[B33] Glading A. Uberall F. Keyse S. M. Lauffenburger D. A. Wells A. (2001). Membrane proximal ERK signaling is required for M-calpain activation downstream of epidermal growth factor receptor signaling. J. Biol. Chem. 276, 23341–23348. 10.1074/jbc.M008847200 11319218

[B34] Gonzalez-Pena D. Nixon S. E. O'Connor J. C. Southey B. R. Lawson M. A. McCusker R. H.. (2016). Microglia Transcriptome Changes in a Model of Depressive Behavior after Immune Challenge. PLoS ONE 11:e0150858. 10.1371/journal.pone.0150858 26959683 PMC4784788

[B35] Guenet J. L. (2005). The mouse genome. Genome Res. 15, 1729–1740. 10.1101/gr.3728305 16339371

[B36] Gutierrez A. P. Lubieniecki K. P. Davidson E. A. Lien S. Kent M. P. Fukui S. (2012). Genetic mapping of quantitative trait loci (QTL) for body-weight in Atlantic salmon (Salmo salar) using a 6.5K SNP array. Aquaculture 358-359, 61–70. 10.1016/j.aquaculture.2012.06.017

[B37] Gutierrez A. P. Yáñez J. M. Fukui S. Swift B. Davidson W. S. (2015). Genome-wide association study (GWAS) for growth rate and age at sexual maturation in Atlantic salmon (Salmo salar). PLoS ONE 10:e0119730. 10.1371/journal.pone.0119730 25757012 PMC4355585

[B38] Habier D. Fernando R. L. Kizilkaya K. Garrick D. J. (2011). Extension of the bayesian alphabet for genomic selection. BMC Bioinformatics 12:186. 10.1186/1471-2105-12-186 21605355 PMC3144464

[B39] Haffray P. Bugeon J. Pincent C. Chapuis H. Mazeiraud E. Rossignol M.-N. (2012). Negative genetic correlations between production traits and head or bony tissues in large all-female rainbow trout (*Oncorhynchus mykiss*). Aquaculture 368–369, 145–152. 10.1016/j.aquaculture.2012.09.023

[B40] Huang D. W. Sherman B. T. Lempicki R. A. (2009a). Bioinformatics enrichment tools: Paths toward the comprehensive functional analysis of large gene lists. Nucleic Acids Res. 37, 1–13. 10.1093/nar/gkn923 19033363 PMC2615629

[B41] Huang D. W. Sherman B. T. Lempicki R. A. (2009b). Systematic and integrative analysis of large gene lists using DAVID bioinformatics resources. Nat. Protoc. 4, 44–57. 10.1038/nprot.2008.211 19131956

[B42] Huttenlocher A. Palecek S. P. Lu Q. Zhang W. Mellgren R. L. Lauffenburger D. A.. (1997). Regulation of cell migration by the calcium-dependent protease calpain. J. Biol. Chem. 272, 32719–32722. 9407041 10.1074/jbc.272.52.32719

[B43] Johnston I. A. Bower N. I. Macqueen D. J. (2011). Growth and the regulation of myotomal muscle mass in teleost fish. J. Exp. Biol. 214, 1617–1628. 10.1242/jeb.038620 21525308

[B44] Kadarmideen H. N. (2014). Genomics to systems biology in animal and veterinary sciences: progress, lessons and opportunities. Livest. Sci. 166, 232–248. 10.1016/j.livsci.2014.04.028

[B45] Kause A. Paananen T. Ritola O. Koskinen H. (2007). Direct and indirect selection of visceral lipid weight, fillet weight, and fillet percentage in a rainbow trout breeding program. J. Anim. Sci. 85, 3218–3227. 10.2527/jas.2007-0332 17709780

[B46] Kause A. Ritola O. Paananen T. Mäntysaari E. Eskelinen U. (2002). Coupling body weight and its composition: a quantitative genetic analysis in rainbow trout. Aquaculture 211, 65–79. 10.1016/S0044-8486(01)00884-5

[B47] Kazi J. U. Kabir N. N. Rönnstrand L. (2015). Role of SRC-like adaptor protein (SLAP) in immune and malignant cell signaling. Cell. Mol. Life Sci. 72, 2535–2544. 10.1007/s00018-015-1882-6 25772501 PMC11113356

[B48] Knight J. D. Kothary R. (2011). The myogenic kinome: protein kinases critical to mammalian skeletal myogenesis. Skelet. Muscle 1:29. 10.1186/2044-5040-1-29 21902831 PMC3180440

[B49] Kocmarek A. L. Ferguson M. M. Danzmann R. G. (2015). Co-localization of growth QTL with differentially expressed candidate genes in rainbow trout. Genome 58, 393–403. 10.1139/gen-2015-0047 26360524

[B50] Konecna A. Frischknecht R. Kinter J. Ludwig A. Steuble M. Meskenaite V.. (2006). Calsyntenin-1 docks vesicular cargo to kinesin-1. Mol. Biol. Cell 17, 3651–3663. 10.1091/mbc.E06-02-0112 16760430 PMC1525238

[B51] Koohmaraie M. (1992). The role of Ca2+-dependent proteases (calpains) in post mortem proteolysis and meat tenderness. Biochimie 74, 239–245. 10.1016/0300-9084(92)90122-U 1610937

[B52] Lee S.-J. Reed L. A. Davies M. V. Girgenrath S. Goad M. E. P. Tomkinson K. N.. (2005). Regulation of muscle growth by multiple ligands signaling through activin type II receptors. Proc. Natl. Acad. Sci. U.S.A. 102, 18117–18122. 10.1073/pnas.0505996102 16330774 PMC1306793

[B53] Leeds T. D. Roger L. V. Weber G. M. Gonzalez-Pena D. Silverstein J. T. (2016). Response to five generations of selection for growth performance traits in rainbow trout (*Oncorhynchus mykiss*) Aquaculture 465, 341–351. 10.1016/j.aquaculture.2016.08.036

[B54] Liu S. Vallejo R. L. Gao G. Palti Y. Weber G. M. Hernandez A.. (2015a). Identification of single-nucleotide polymorphism markers associated with cortisol response to crowding in rainbow trout. Mar. Biotechnol. (NY). 17, 328–337. 10.1007/s10126-015-9621-4 25652693

[B55] Liu S. Vallejo R. L. Palti Y. Gao G. Marancik D. P. Hernandez A. G.. (2015b). Identification of single nucleotide polymorphism markers associated with bacterial cold water disease resistance and spleen size in rainbow trout. Front. Genet. 6:298. 10.3389/fgene.2015.00298 26442114 PMC4585308

[B56] Martin A. Ochagavia M. E. Rabasa L. C. Miranda J. Fernandez-de-Cossio J. Bringas R. (2010). BisoGenet: a new tool for gene network building, visualization and analysis. BMC Bioinformatics 11:91. 10.1186/1471-2105-11-91 20163717 PMC3098113

[B57] Meuwissen T. H. E. Hayes B. J. Goddard M. E. (2001). Prediction of total genetic value using genome-wide dense marker maps. Genetics 157, 1819–1829. 11290733 10.1093/genetics/157.4.1819 PMC1461589

[B58] Misztal I. Legarra A. Aguilar I. (2009). Computing procedures for genetic evaluation including phenotypic, full pedigree, and genomic information. J. Dairy Sci. 92, 4648–4655. 10.3168/jds.2009-2064 19700728

[B59] Misztal I. Tsuruta S. Strabel T. Auvray B. Druet T. Lee D. H. (2002). BLUPF90 and related programs (BGF90) [WWW Document], in Proceeding of 7th World Congress on Genetics Applied to Livestock Production (Montpellier), Available online at: http://nce.ads.uga.edu/wiki/lib/exe/fetch.php?media=28-07.pdf (Accessed March 14, 16)

[B60] Mohassel P. Rosen P. Casciola-Rosen L. Pak K. Mammen A. L. (2015). Expression of the dermatomyositis autoantigen transcription intermediary factor 1γ in regenerating muscle. Arthritis Rheumatol. 67, 266–272. 10.1002/art.38863 25186009 PMC4280343

[B61] Mommsen T. P. (2001). Paradigms of growth in fish [WWW Document], in Comparative Biochemistry and Physiology Part B Biochemistry and Molecular Biology. Available online at: https://www.researchgate.net/profile/Thomas_Mommsen/publication_journalabbrev/11941082_Paradigms_of_growth_in_fish/links/0fcfd5092b79849023000000.pdf (Accessed January 22, 16). 10.1016/s1096-4959(01)00312-8 11399452

[B62] Neira R. Lhorente J. P. Araneda C. Díaz N. Bustos E. Alert A. (2004). Studies on carcass quality traits in two populations of Coho salmon (*Oncorhynchus kisutch*): phenotypic and genetic parameters. Aquaculture 241, 117–131. 10.1016/j.aquaculture.2004.08.009

[B63] Nielsen H. M. Sonesson A. K. Yazdi H. Meuwissen T. H. E. (2009). Comparison of accuracy of genome-wide and BLUP breeding value estimates in sib based aquaculture breeding schemes. Aquaculture 289, 259–264. 10.1016/j.aquaculture.2009.01.027

[B64] O'Malley K. G. Sakamoto T. Danzmann R. G. Ferguson M. M. (2003). Quantitative trait loci for spawning date and body weight in rainbow trout: testing for conserved effects across ancestrally duplicated chromosomes. J. Hered. 94, 273–284. 10.1093/jhered/esg067 12920098

[B65] Odegård J. Moen T. Santi N. Korsvoll S. A. Kjøglum S. Meuwissen T. H. E. (2014). Genomic prediction in an admixed population of Atlantic salmon (Salmo salar). Front. Genet. 5:402. 10.3389/fgene.2014.00402 25484890 PMC4240172

[B66] Ortiz-Medina H. Emond M. R. Jontes J. D. (2015). Zebrafish calsyntenins mediate homophilic adhesion through their amino-terminal cadherin repeats. Neuroscience 286, 87–96. 10.1016/j.neuroscience.2014.11.030 25463516 PMC4298480

[B67] Ory S. Zhou M. Conrads T. P. Veenstra T. D. Morrison D. K. (2003). Protein phosphatase 2A positively regulates Ras signaling by dephosphorylating KSR1 and Raf-1 on critical 14-3-3 binding sites. Curr. Biol. 13, 1356–1364. 10.1016/S0960-9822(03)00535-9 12932319

[B68] Palti Y. Gao G. Liu S. Kent M. P. Lien S. Miller M. R.. (2015a). The development and characterization of a 57K single nucleotide polymorphism array for rainbow trout. Mol. Ecol. Resour. 15, 662–672. 10.1111/1755-0998.12337 25294387

[B69] Palti Y. Vallejo R. L. Gao G. Liu S. Hernandez A. G. Rexroad C. E.. (2015b). Detection and validation of QTL affecting bacterial cold water disease resistance in rainbow trout using restriction-site associated DNA sequencing. PLoS ONE 10:e0138435. 10.1371/journal.pone.0138435 26376182 PMC4574402

[B70] Patel Y. M. Lane M. D. (2000). Mitotic clonal expansion during preadipocyte differentiation: calpain-mediated turnover of p27. J. Biol. Chem. 275, 17653–17660. 10.1074/jbc.M910445199 10749891

[B71] Perry G. M. L. Ferguson M. M. Sakamoto T. Danzmann R. G. (2005). Sex-linked quantitative trait loci for thermotolerance and length in the rainbow trout. J. Hered. 96, 97–107. 10.1093/jhered/esi019 15653562

[B72] Phelps M. P. Jaffe I. M. Bradley T. M. (2013). Muscle growth in teleost fish is regulated by factors utilizing the activin II B receptor. J. Exp. Biol. 216, 3742–3750. 10.1242/jeb.086660 23788712

[B73] Ponomareva O. Y. Holmen I. C. Sperry A. J. Eliceiri K. W. Halloran M. C. (2014). Calsyntenin-1 regulates axon branching and endosomal trafficking during sensory neuron development *in vivo*. J. Neurosci. 34, 9235–9248. 10.1523/JNEUROSCI.0561-14.2014 25009257 PMC4087204

[B74] Potter D. A. Tirnauer J. S. Janssen R. Croall D. E. Hughes C. N. Fiacco K. A.. (1998). Calpain regulates actin remodeling during cell spreading. J. Cell Biol. 141, 647–662. 9566966 10.1083/jcb.141.3.647 PMC2132736

[B75] Powell J. White I. Guy D. Brotherstone S. (2008). Genetic parameters of production traits in Atlantic salmon (Salmo salar). Aquaculture 274, 225–231. 10.1016/j.aquaculture.2007.11.036

[B76] Rastas P. Paulin L. Hanski I. Lehtonen R. Auvinen P. (2013). Lep-MAP: fast and accurate linkage map construction for large SNP datasets. Bioinformatics 29, 3128–3134. 10.1093/bioinformatics/btt563 24078685 PMC4433499

[B77] Sadkowski T. Ciecierska A. Majewska A. Oprządek J. Dasiewicz K. Ollik M.. (2014). Transcriptional background of beef marbling - novel genes implicated in intramuscular fat deposition. Meat Sci. 97, 32–41. 10.1016/j.meatsci.2013.12.017 24491505

[B78] Sae-Lim P. Komen H. Kause A. van Arendonk J. A. M. Barfoot A. J. Martin K. E.. (2012). Defining desired genetic gains for rainbow trout breeding objective using analytic hierarchy process. J. Anim. Sci. 90, 1766–1776. 10.2527/jas.2011-4267 22178851

[B79] Salem M. Nath J. Rexroad C. E. Killefer J. Yao J. (2005). Identification and molecular characterization of the rainbow trout calpains (Capn1 and Capn2): their expression in muscle wasting during starvation. Comp. Biochem. Physiol. B Biochem. Mol. Biol. 140, 63–71. 10.1016/j.cbpc.2004.09.007 15621511

[B80] Salmerón C. Navarro I. Johnston I. A. Gutiérrez J. Capilla E. (2015). Characterisation and expression analysis of cathepsins and ubiquitin-proteasome genes in gilthead sea bream (*Sparus aurata*) skeletal muscle. BMC Res. Notes 8:149. 10.1186/s13104-015-1121-0 25880457 PMC4431372

[B81] Sato K. Kawashima S. (2001). Calpain function in the modulation of signal transduction molecules. Biol. Chem. 382, 743–751. 10.1515/BC.2001.090 11517927

[B82] Seiliez I. Sabin N. Gabillard J.-C. (2012). Myostatin inhibits proliferation but not differentiation of trout myoblasts. Mol. Cell. Endocrinol. 351, 220–226. 10.1016/j.mce.2011.12.011 22209759

[B83] Serão N. V. González-Peña D. Beever J. E. Faulkner D. B. Southey B. R. Rodriguez-Zas S. L. (2013). Single nucleotide polymorphisms and haplotypes associated with feed efficiency in beef cattle. BMC Genet. 14:94. 10.1186/1471-2156-14-94 24066663 PMC3819741

[B84] Shannon P. Markiel A. Ozier O. Baliga N. S. Wang J. T. Ramage D.. (2003). Cytoscape: a software environment for integrated models of biomolecular interaction networks. Genome Res. 13, 2498–2504. 10.1101/gr.1239303 14597658 PMC403769

[B85] Sharma A. Lee J. S. Dang C. G. Sudrajad P. Kim H. C. Yeon S. H.. (2015). Stories and challenges of genome wide association studies in livestock - a review. Asian Aus. J. Anim. Sci. 28, 1371–1379. 10.5713/ajas.14.0715 26194229 PMC4554843

[B86] Sosinowski T. Killeen N. Weiss A. (2001). The Src-like adaptor protein downregulates the T cell receptor on CD4+CD8+ thymocytes and regulates positive selection. Immunity 15, 457–466. 10.1016/S1074-7613(01)00195-9 11567635

[B87] Sun X. Habier D. Fernando R. L. Garrick D. J. Dekkers J. C. (2011). Genomic breeding value prediction and QTL mapping of QTLMAS2010 data using Bayesian Methods. BMC Proc. 5 (Suppl. 3):S13. 10.1186/1753-6561-5-S3-S13 21624169 PMC3103198

[B88] Takahashi K. Yamanaka S. (2006). Induction of pluripotent stem cells from mouse embryonic and adult fibroblast cultures by defined factors. Cell 126, 663–676. 10.1016/j.cell.2006.07.024 16904174

[B89] Tsai H.-Y. Hamilton A. Tinch A. E. Guy D. R. Gharbi K. Stear M. J.. (2015). Genome wide association and genomic prediction for growth traits in juvenile farmed Atlantic salmon using a high density SNP array. BMC Genomics 16:969. 10.1186/s12864-015-2117-9 26582102 PMC4652364

[B90] Turner S. D. (2014). qqman: an R package for visualizing GWAS results using Q-Q and manhattan plots. bioRxiv. 10.1101/005165

[B91] Van't Padje S. Chaudhry B. Severijnen L.-A. van der Linde H. C. Mientjes E. J. Oostra B. A.. (2009). Reduction in fragile X related 1 protein causes cardiomyopathy and muscular dystrophy in zebrafish. J. Exp. Biol. 212, 2564–2570. 10.1242/jeb.032532 19648401

[B92] VanRaden P. M. (2008). Efficient methods to compute genomic predictions. J. Dairy Sci. 91, 4414–4423. 10.3168/jds.2007-0980 18946147

[B93] Wang H. Misztal I. Aguilar I. Legarra A. Fernando R. L. Vitezica Z.. (2014). Genome-wide association mapping including phenotypes from relatives without genotypes in a single-step (ssGWAS) for 6-week body weight in broiler chickens. Front. Genet. 5:134. 10.3389/fgene.2014.00134 24904635 PMC4033036

[B94] Wang H. Misztal I. Aguilar I. Legarra A. Muir W. M. (2012). Genome-wide association mapping including phenotypes from relatives without genotypes. Genet. Res. (Camb). 94, 73–83. 10.1017/S0016672312000274 22624567

[B95] Wringe B. F. Devlin R. H. Ferguson M. M. Moghadam H. K. Sakhrani D. Danzmann R. G. (2010). Growth-related quantitative trait loci in domestic and wild rainbow trout (*Oncorhynchus mykiss*). BMC Genet. 11:63. 10.1186/1471-2156-11-63 20609225 PMC2914766

[B96] Xi Q. Wang Z. Zaromytidou A.-I. Zhang X. H.-F. Chow-Tsang L.-F. Liu J. X.. (2011). A poised chromatin platform for TGF-β access to master regulators. Cell 147, 1511–1524. 10.1016/j.cell.2011.11.032 22196728 PMC3582033

[B97] Yáñez J. M. Newman S. Houston R. D. (2015). Genomics in aquaculture to better understand species biology and accelerate genetic progress. Front. Genet. 6:128. 10.3389/fgene.2015.00128 25883603 PMC4381651

[B98] Zhang S. Cui W. (2014). Sox2, a key factor in the regulation of pluripotency and neural differentiation. World J. Stem Cells 6, 305–311. 10.4252/wjsc.v6.i3.305 25126380 PMC4131272

[B99] Zhang X. Lourenco D. Aguilar I. Legarra A. Misztal I. (2016). Weighting strategies for single-step genomic BLUP: an iterative approach for accurate calculation of GEBV and GWAS. Front. Genet. 7:151. 10.3389/fgene.2016.00151 27594861 PMC4990542

