# Supplementary material for: Genome-Wide Association Study for Identifying Loci that Affect Fillet Yield, Carcass, and Body Weight Traits in Rainbow Trout (Oncorhynchus mykiss)
Source: Front Genet. 2016 Nov 22;7:203. doi: 10.3389/fgene.2016.00203 (PMC5118429; doi:10.3389/fgene.2016.00203)
Supplement: Table S3 — The 40 SNP markers from the two windows that explained the largest proportion of variance for FW and harboring or neighboring genes from the same genome scaffold (Berthelot et al., 2014). [file Table3.DOCX]

**S3 Table.** The 40 SNP markers from the two windows that explained the largest proportion of variance for fillet weight and harboring or neighboring genes from the same genome scaffold (Berthelot et al., 2014).

| Marker | Chr | Position (cM) | Alleles | VE (%) | Scaffold number | Scaffold Position | Scaffold Size | Location | Description |
| --- | --- | --- | --- | --- | --- | --- | --- | --- | --- |
| **Window 1 Total proportion 1.2%** | | | | | | | | | |
| AX-89948616 | 9 | 125.19 | C/A | 0.00 | scaffold_516 | 471807 | 728099 | Intron | Pol polyprotein |
| AX-89937787 | 9 | 125.19 | G/T | 0.01 | scaffold_52 | 2118742 | 2128772 | Exon 6 | E3 ubiquitin-protein ligase trim33 |
| AX-89976492 | 9 | 125.19 | T/C | 0.12 | scaffold_8612 | 10780 | 26627 | Intron | Beta-catenin-interacting protein 1 isoform x1 |
| AX-89970327 | 9 | 125.19 | A/C | 0.10 | scaffold_516 | 399414 | 728099 | Intron | Calsyntenin-1-like isoform x2 |
| AX-89940136 | 9 | 125.19 | A/G | 0.09 | scaffold_516 | 407663 | 728099 | Intron | Calsyntenin-1-like isoform x2 |
| AX-89936139 | 9 | 125.67 | A/C | 0.11 |  |  |  | Near* | None |
| AX-89944669 | 9 | 125.67 | G/T | 0.11 | scaffold_32707 | 921 | 3946 | Exon 1 | Nudix hydrolase chloroplastic-like |
| AX-89940514 | 9 | 125.67 | T/C | 0.10 | scaffold_10308 | 15430 | 21663 | Near* | None |
| AX-89937939 | 9 | 125.67 | A/C | 0.06 | scaffold_52 | 1871366 | 2128772 | Near* | Otu domain-containing protein 3 **/**  Von willebrand factor a domain-containing protein 1-like |
| AX-89937961 | 9 | 125.67 | G/T | 0.11 | scaffold_516 | 586177 | 728099 | Exon 6 | Properdin |
| AX-89951506 | 9 | 125.67 | G/A | 0.11 | scaffold_516 | 555991 | 728099 | Exon 3 | Transmembrane protein 201-like |
| AX-89936264 | 9 | 125.67 | A/C | 0.04 | scaffold_52 | 2058649 | 2128772 | Intron | Denn domain-containing protein 2a |
| AX-89942568 | 9 | 125.67 | C/T | 0.00 | scaffold_9849 | 19614 | 22834 | Near* | None |
| AX-89943050 | 9 | 125.86 | G/A | 0.06 | scaffold_52 | 1797105 | 2128772 | Intron | Atpase family aaa domain-containing protein 3-like |
| AX-89923874 | 9 | 125.86 | G/A | 0.03 | scaffold_52 | 1913655 | 2128772 | Near* | Von willebrand factor a domain-containing protein 1-like **/** Transmembrane and coiled-coil domain-containing protein 4 |
| AX-89938525 | 9 | 125.86 | G/A | 0.07 | scaffold_52 | 1795215 | 2128772 | Intron | Atpase family aaa domain-containing protein 3-like |
| AX-89943624 | 9 | 125.86 | G/A | 0.01 | scaffold_52 | 2127868 | 2128772 | Exon 10 | Lysosomal amino acid transporter 1 homolog |
| AX-89957923 | 9 | 126.04 | A/G | 0.07 | scaffold_19674 | 6079 | 8261 | Near* | None |
| AX-89977046 | 9 | 126.21 | A/G | 0.00 | scaffold_8044 | 5880 | 28838 | Intron | Anoctamin-7-like |
| AX-89920792 | 9 | 126.24 | C/T | 0.00 | scaffold_32638 | 2560 | 3953 | Exon 1 | Rho guanine nucleotide exchange factor 19-like |
| **Window 2 Total proportion 1.1%** | | | | | | | | | |
| AX-89965882 | 9 | 116.06 | C/T | 0.01 | scaffold_1609 | 154586 | 200099 | Near* | Serine arginine repetitive matrix protein 2-like **/**  Kelch domain-containing protein 8b |
| AX-89953042 | 9 | 116.09 | G/A | 0.10 | scaffold_1609 | 166168 | 200099 | Exon 4 | Kelch domain-containing protein 8b |
| AX-89945535 | 9 | 116.21 | T/G | 0.03 | scaffold_589 | 626284 | 646930 | Near* | Kelch domain-containing protein 8b-like **/** None |
| AX-89947998 | 9 | 116.36 | C/T | 0.05 | scaffold_347 | 916340 | 935129 | Exon 3 | Mrg-binding protein |
| AX-89953467 | 9 | 116.43 | C/A | 0.02 | scaffold_347 | 581759 | 935129 | Intron | Extracellular sulfatase sulf-2 isoform x2 |
| AX-89947184 | 9 | 116.43 | G/T | 0.00 |  |  |  | Near* | None |
| AX-89931050 | 9 | 116.43 | G/A | 0.01 | scaffold_26318 | 3116 | 5070 | Near* | None |
| AX-89926731 | 9 | 116.43 | A/G | 0.01 | scaffold_347 | 41933 | 935129 | Exon 2 | Potassium voltage-gated channel subfamily d member 1-like |
| AX-89977034 | 9 | 116.43 | G/T | 0.04 | scaffold_347 | 796372 | 935129 | Near* | Extracellular sulfatase sulf-2 **/**  Mrg-binding protein |
| AX-89951447 | 9 | 117.12 | G/A | 0.13 | scaffold_43535 | 864 | 3113 | Near* | None |
| AX-89928960 | 9 | 117.12 | T/C | 0.05 | scaffold_347 | 257504 | 935129 | Exon 6 | Serine threonine-protein kinase pim-1 |
| AX-89928957 | 9 | 117.12 | C/T | 0.06 | scaffold_347 | 580051 | 935129 | Exon 11 | Extracellular sulfatase sulf-2 isoform x2 |
| AX-89917535 | 9 | 117.12 | G/T | 0.04 | scaffold_347 | 580189 | 935129 | Intron | Extracellular sulfatase sulf-2 isoform x2 |
| AX-89975284 | 9 | 117.12 | A/C | 0.11 | scaffold_347 | 348224 | 935129 | Exon 7 | Src-like-adapter 2 |
| AX-89940159 | 9 | 117.12 | T/C | 0.12 | scaffold_347 | 148757 | 935129 | Intron | Serine threonine-protein kinase sbk1-like |
| AX-89955606 | 9 | 117.12 | C/T | 0.03 |  |  |  | Near* | None |
| AX-89925202 | 9 | 117.29 | T/G | 0.04 | scaffold_347 | 422258 | 935129 | Intron | Phd finger protein 20-like isoform x3 |
| AX-89929243 | 9 | 117.91 | T/C | 0.08 | scaffold_347 | 256419 | 935129 | Exon 4 | Serine threonine-protein kinase pim-1 |
| AX-89929764 | 9 | 117.91 | C/T | 0.07 | scaffold_347 | 305735 | 935129 | Near* | Glutathione synthetase-like **/**  Myosin-7b |
| AX-89924961 | 9 | 117.91 | C/T | 0.13 | scaffold_347 | 414782 | 935129 | Exon 6 | Phd finger protein 20-like isoform x3 |

Chr: chromosome; VE: percentage of the genetic variance explained by the SNP.
