# Supplementary material for: Genome-Wide Association Study for Identifying Loci that Affect Fillet Yield, Carcass, and Body Weight Traits in Rainbow Trout (Oncorhynchus mykiss)
Source: Front Genet. 2016 Nov 22;7:203. doi: 10.3389/fgene.2016.00203 (PMC5118429; doi:10.3389/fgene.2016.00203)
Supplement: Table S4 — The 20 SNP markers from the one window that explained the largest proportion of variance for BW10 and harboring or neighboring genes from the same genome scaffold (Berthelot et al., 2014). [file Table4.DOCX]

**S4 Table.** The 20 SNP markers from the one window that explained the largest proportion of variance for 10-month body weight and harboring or neighboring genes from the same genome scaffold (Berthelot et al., 2014).

| Marker | Chr | Position (cM) | Alleles | VE (%) | Scaffold number | Scaffold Position | Scaffold Size | Location | Description |
| --- | --- | --- | --- | --- | --- | --- | --- | --- | --- |
| **Window 1 Total proportion 1.4%** | | | | | | | | | |
| AX-89917954 | 5 | 128.48 | T/G | 0.08 | scaffold_1354 | 226414 | 256005 | Intron | ankyrin repeat and mynd domain-containing protein 1 |
| AX-89955131 | 5 | 128.48 | G/A | 0.08 | scaffold_1354 | 235530 | 256005 | Exon9 | glypican-1-like |
| AX-89924134 | 5 | 128.55 | G/T | 0.07 | scaffold_133 | 1407613 | 1550032 | Near | fragile x mental retardation syndrome-related protein 1-like isoform x5/tetratricopeptide repeat protein 14 |
| AX-89968382 | 5 | 128.55 | C/T | 0.07 | scaffold_133 | 1437875 | 1550032 | Exon2 | leucine-rich repeat-containing protein 24-like |
| AX-89935999 | 5 | 128.55 | C/T | 0.02 | scaffold_1354 | 54654 | 256005 | Intron | ephrin type-b receptor 1 |
| AX-89951908 | 5 | 128.55 | C/A | 0.02 | scaffold_133 | 1242064 | 1550032 | Exon1 | transcription factor sox-2 |
| AX-89960561 | 5 | 128.55 | G/A | 0.09 | scaffold_133 | 1367963 | 1550032 | Intron | fragile x mental retardation syndrome-related protein 1-like isoform x5 |
| AX-89975289 | 5 | 128.55 | G/A | 0.04 | scaffold_133 | 1495514 | 1550032 | Near | leucine-rich repeat-containing protein 24-like/None |
| AX-89940409 | 5 | 128.55 | G/A | 0.05 | scaffold_1354 | 129122 | 256005 | Near | transposable element tcb1 transposase/pinopsin- partial |
| AX-89942374 | 5 | 128.55 | G/T | 0.07 | scaffold_133 | 1348138 | 1550032 | Near | transcription factor sox-2/mitochondrial import inner membrane translocase subunit tim14 |
| AX-89947679 | 5 | 128.55 | C/A | 0.08 | scaffold_133 | 1242004 | 1550032 | Exon1 | transcription factor sox-2 |
| AX-89937568 | 5 | 128.55 | C/T | 0.08 | scaffold_133 | 1352168 | 1550032 | Intron | mitochondrial import inner membrane translocase subunit tim14 |
| AX-89950855 | 5 | 128.55 | T/C | 0.07 | scaffold_133 | 1243876 | 1550032 | Near | transcription factor sox-2/mitochondrial import inner membrane translocase subunit tim14 |
| AX-89967654 | 5 | 128.55 | A/C | 0.08 | scaffold_133 | 1186795 | 1550032 | Near | leucine-rich repeat-containing protein 40/transcription factor sox-2 |
| AX-89938437 | 5 | 128.55 | G/A | 0.08 | scaffold_42527 | 1677 | 3171 | Near | None/None |
| AX-89932385 | 5 | 128.55 | G/A | 0.08 | scaffold_133 | 1186941 | 1550032 | Near | leucine-rich repeat-containing protein 40/transcription factor sox-2 |
| AX-89970155 | 5 | 128.55 | C/T | 0.08 | scaffold_133 | 1334585 | 1550032 | Near | transcription factor sox-2/mitochondrial import inner membrane translocase subunit tim14 |
| AX-89942169 | 5 | 128.55 | A/G | 0.08 | scaffold_133 | 1297202 | 1550032 | Near | transcription factor sox-2/mitochondrial import inner membrane translocase subunit tim14 |
| AX-89933877 | 5 | 128.55 | C/A | 0.08 | scaffold_133 | 1451999 | 1550032 | Near | leucine-rich repeat-containing protein 24-like/None |
| AX-89944791 | 5 | 128.55 | T/C | 0.08 | scaffold_133 | 1169256 | 1550032 | Near | leucine-rich repeat-containing protein 40/transcription factor sox-2 |

Chr: chromosome; VE: percentage of the genetic variance explained by the SNP.
