# Supplementary material for: Genome-Wide Association Study for Identifying Loci that Affect Fillet Yield, Carcass, and Body Weight Traits in Rainbow Trout (Oncorhynchus mykiss)
Source: Front Genet. 2016 Nov 22;7:203. doi: 10.3389/fgene.2016.00203 (PMC5118429; doi:10.3389/fgene.2016.00203)
Supplement: Table S6 — The 60 SNP markers from the three windows that explained the largest proportion of variance for CAR and harboring or neighboring genes from the same genome scaffold (Berthelot et al., 2014). [file Table6.DOCX]

**S6 Table.** The 60 SNP markers from the three windows that explained the largest proportion of variance for carcass weight and harboring or neighboring genes from the same genome scaffold (Berthelot et al., 2014).

| Marker | Chr | Position (cM) | Alleles | VE (%) | Scaffold number | Scaffold Position | Scaffold Size | Location | Description |
| --- | --- | --- | --- | --- | --- | --- | --- | --- | --- |
| **Window 1 Total proportion 1.7%** | | | | | | | | | |
| AX-89963727 | 27 | 75.09 | A/G | 0.08 | scaffold_5094 | 43315 | 45303 | Near | None |
| AX-89952551 | 27 | 75.09 | A/G | 0.12 | scaffold_1006 | 143453 | 383627 | Near | nitric oxide inducible / serine threonine-protein kinase nlk |
| AX-89957877 | 27 | 75.09 | C/T | 0.00 | scaffold_1006 | 34540 | 383627 | Exon5 | 28s ribosomal protein mitochondrial-like |
| AX-89954149 | 27 | 75.09 | C/A | 0.12 | scaffold_147 | 921922 | 1497438 | Near | atp-sensitive inward rectifier potassium channel 1-like / cmp-n-acetylneuraminate-beta-galactosamide-alpha- -sialyltransferase 4-like isoform x1 |
| AX-89938133 | 27 | 75.09 | A/G | 0.13 | scaffold_1006 | 46265 | 383627 | Exon3 | nitric oxide inducible |
| AX-89948564 | 27 | 74.78 | G/A | 0.12 | scaffold_8798 | 5532 | 26005 | Near | Undetermined / None |
| AX-89931282 | 27 | 74.76 | C/T | 0.01 | scaffold_30488 | 3000 | 4228 | Near | None |
| AX-89974542 | 27 | 74.58 | G/T | 0.11 | scaffold_842 | 38757 | 463739 | Intron | kinase suppressor of ras 1-like isoform x2 |
| AX-89973258 | 27 | 74.58 | C/T | 0.00 | scaffold_33132 | 426 | 3898 | Near | None |
| AX-89926230 | 27 | 74.58 | A/G | 0.12 | scaffold_1952 | 116665 | 147230 | Near | neurofibromin isoform x2 / oligodendrocyte-myelin glyco |
| AX-89938965 | 27 | 74.43 | G/T | 0.12 | scaffold_842 | 38933 | 463739 | Intron | kinase suppressor of ras 1-like isoform x2 |
| AX-89922469 | 27 | 73.96 | G/A | 0.09 | scaffold_20938 | 5251 | 7716 | Near | None |
| AX-89928353 | 27 | 73.96 | G/A | 0.10 | scaffold_1675 | 177158 | 191261 | Intron | vascular endothelial zinc finger 1-like isoform x2 |
| AX-89968747 | 27 | 73.96 | A/G | 0.11 | scaffold_3611 | 60104 | 62580 | Intron | unconventional myosin-xviiia-like isoform x1 |
| AX-89922302 | 27 | 73.96 | A/G | 0.04 |  |  |  | Near | None |
| AX-89947091 | 27 | 73.96 | na | 0.10 |  |  |  | na | na |
| AX-89922699 | 27 | 73.96 | T/C | 0.08 | scaffold_20938 | 1975 | 7716 | Near | None |
| AX-89972785 | 27 | 73.96 | C/T | 0.06 | scaffold_33132 | 229 | 3898 | Near | None |
| AX-89923476 | 27 | 73.7 | G/T | 0.03 | scaffold_1952 | 65855 | 147230 | Near | a-kinase anchor protein mitochondrial-like / neurofibromin isoform x2 |
| AX-89942611 | 27 | 73.42 | C/A | 0.16 | scaffold_3980 | 33385 | 57002 | Intron | unconventional myosin-xviiia-like isoform x2 |
| **Window 2 Total proportion 1.7%** | | | | | | | | | |
| AX-89943160 | 17 | 116.45 | A/G | 0.06 | scaffold_24 | 484345 | 2579057 | Intron | echinoderm microtubule-associated 6 |
| AX-89932584 | 17 | 116.45 | C/T | 0.04 | scaffold_24 | 627613 | 2579057 | Exon18 | ribosome-binding protein 1 isoform x1 |
| AX-89945639 | 17 | 115.87 | G/T | 0.03 | scaffold_24 | 311846 | 2579057 | Exon2 | 3-hydroxybutyrate dehydrogenase type 2 |
| AX-89960063 | 17 | 115.87 | A/G | 0.08 | scaffold_24 | 625943 | 2579057 | Exon19 | ribosome-binding protein 1 isoform x1 |
| AX-89973675 | 17 | 115.87 | G/T | 0.10 | scaffold_26752 | 3396 | 4948 | Intron | spectrin beta non-erythrocytic 1-like |
| AX-89969415 | 17 | 115 | G/A | 0.08 | scaffold_24 | 153478 | 2579057 | Near | splicing factor 3a subunit 2-like / calpain-1 catalytic subunit-like |
| AX-89940276 | 17 | 115 | C/A | 0.05 | scaffold_24 | 153428 | 2579057 | Near | splicing factor 3a subunit 2-like / calpain-1 catalytic subunit-like |
| AX-89974461 | 17 | 115 | T/G | 0.03 | scaffold_24 | 374273 | 2579057 | Intron | loc100135362 protein |
| AX-89969602 | 17 | 114.85 | C/T | 0.10 | scaffold_24 | 197378 | 2579057 | Intron | calpain-2 catalytic subunit-like |
| AX-89976360 | 17 | 114.44 | G/A | 0.09 | scaffold_24 | 54552 | 2579057 | Intron | transmembrane protein 63b-like isoform x2 |
| AX-89945709 | 17 | 114.44 | A/C | 0.09 | scaffold_24 | 39900 | 2579057 | Intron | transmembrane protein 63b-like isoform x2 |
| AX-89934222 | 17 | 114.44 | G/A | 0.09 | scaffold_24 | 54476 | 2579057 | Intron | transmembrane protein 63b-like isoform x2 |
| AX-89952148 | 17 | 114.19 | G/A | 0.09 | scaffold_24 | 21794 | 2579057 | Near | None / transmembrane protein 63b-like isoform x2 |
| AX-89935000 | 17 | 114.02 | A/G | 0.17 | scaffold_173 | 119365 | 1392108 | Near | histone h2b 1 2-like / ubiquitin-conjugating enzyme e2 variant 1 |
| AX-89918454 | 17 | 114.02 | C/A | 0.12 | scaffold_173 | 123912 | 1392108 | Near | histone h2b 1 2-like / ubiquitin-conjugating enzyme e2 variant 1 |
| AX-89968768 | 17 | 113.9 | G/T | 0.00 | scaffold_24 | 40885 | 2579057 | Intron | transmembrane protein 63b-like isoform x2 |
| AX-89923840 | 17 | 113.51 | C/T | 0.18 | scaffold_173 | 195297 | 1392108 | Exon4 | ubiquitin-conjugating enzyme e2 variant 1 |
| AX-89954166 | 17 | 113.51 | T/G | 0.03 | scaffold_173 | 285467 | 1392108 | Intron | solute carrier family facilitated glucose transporter member 10 |
| AX-89925576 | 17 | 113.51 | A/G | 0.18 | scaffold_173 | 160398 | 1392108 | Near | histone h2b 1 2-like / ubiquitin-conjugating enzyme e2 variant 1 |
| AX-89944198 | 17 | 113.44 | G/T | 0.02 | scaffold_173 | 307865 | 1392108 | Intron | serine incorporator 1-like |
| **Window 3 Total proportion 1.0%** | | | | | | | | | |
| AX-89948616 | 9 | 125.19 | C/A | 0.00 | scaffold_516 | 471807 | 728099 | Intron | Pol polyprotein |
| AX-89937787 | 9 | 125.19 | G/T | 0.02 | scaffold_52 | 2118742 | 2128772 | Exon 6 | E3 ubiquitin-protein ligase trim33 |
| AX-89976492 | 9 | 125.19 | T/C | 0.08 | scaffold_8612 | 10780 | 26627 | Intron | Beta-catenin-interacting protein 1 isoform x1 |
| AX-89970327 | 9 | 125.19 | A/C | 0.08 | scaffold_516 | 399414 | 728099 | Intron | Calsyntenin-1-like isoform x2 |
| AX-89940136 | 9 | 125.19 | A/G | 0.10 | scaffold_516 | 407663 | 728099 | Intron | Calsyntenin-1-like isoform x2 |
| AX-89936139 | 9 | 125.67 | A/C | 0.08 | 0 | 0 | 0 | Near* | None |
| AX-89944669 | 9 | 125.67 | G/T | 0.08 | scaffold_32707 | 921 | 3946 | Exon 1 | Nudix hydrolase chloroplastic-like |
| AX-89940514 | 9 | 125.67 | T/C | 0.05 | scaffold_10308 | 15430 | 21663 | Near* | None |
| AX-89937939 | 9 | 125.67 | A/C | 0.02 | scaffold_52 | 1871366 | 2128772 | Near* | Otu domain-containing protein 3 **/**  Von willebrand factor a domain-containing protein 1-like |
| AX-89937961 | 9 | 125.67 | G/T | 0.08 | scaffold_516 | 586177 | 728099 | Exon 6 | Properdin |
| AX-89951506 | 9 | 125.67 | G/A | 0.08 | scaffold_516 | 555991 | 728099 | Exon 3 | Transmembrane protein 201-like |
| AX-89936264 | 9 | 125.67 | A/C | 0.01 | scaffold_52 | 2058649 | 2128772 | Intron | Denn domain-containing protein 2a |
| AX-89942568 | 9 | 125.67 | C/T | 0.00 | scaffold_9849 | 19614 | 22834 | Near* | None |
| AX-89943050 | 9 | 125.86 | G/A | 0.09 | scaffold_52 | 1797105 | 2128772 | Intron | Atpase family aaa domain-containing protein 3-like |
| AX-89923874 | 9 | 125.86 | G/A | 0.02 | scaffold_52 | 1913655 | 2128772 | Near* | Von willebrand factor a domain-containing protein 1-like **/** Transmembrane and coiled-coil domain-containing protein 4 |
| AX-89938525 | 9 | 125.86 | G/A | 0.09 | scaffold_52 | 1795215 | 2128772 | Intron | Atpase family aaa domain-containing protein 3-like |
| AX-89943624 | 9 | 125.86 | G/A | 0.05 | scaffold_52 | 2127868 | 2128772 | Exon 10 | Lysosomal amino acid transporter 1 homolog |
| AX-89957923 | 9 | 126.04 | A/G | 0.06 | scaffold_19674 | 6079 | 8261 | Near* | None |
| AX-89977046 | 9 | 126.21 | A/G | 0.02 | scaffold_8044 | 5880 | 28838 | Intron | Anoctamin-7-like |
| AX-89920792 | 9 | 126.24 | C/T | 0.00 | scaffold_32638 | 2560 | 3953 | Exon 1 | Rho guanine nucleotide exchange factor 19-like |

Chr: chromosome; VE: percentage of the genetic variance explained by the SNP.
